# A Scientometric Review of Alexithymia: Mapping Thematic and Disciplinary Shifts in Half a Century of Research

**DOI:** 10.3389/fpsyt.2020.611489

**Published:** 2020-12-10

**Authors:** Giulia Gaggero, Andrea Bonassi, Sara Dellantonio, Luigi Pastore, Vahid Aryadoust, Gianluca Esposito

**Affiliations:** ^1^Department of Psychology and Cognitive Science, University of Trento, Rovereto, Italy; ^2^Mobile and Social Computing Lab, Bruno Kessler Foundation, Trento, Italy; ^3^Department of Education, Psychology, Communication, University of Bari, Bari, Italy; ^4^National Institute of Education, Nanyang Technological University, Singapore, Singapore; ^5^Psychology Program, School of Social Sciences, Nanyang Technological University, Singapore, Singapore; ^6^Lee Kong Chian School of Medicine, Nanyang Technological University, Singapore, Singapore

**Keywords:** alexithymia, co-citation analysis, affect regulation, science mapping, citespace, systematic review, emotional processing, scientometric review

## Abstract

The term “alexithymia” was introduced in the lexicon of psychiatry in the early ‘70s by Sifneos to outline the difficulties manifested by some patients in identifying and describing their own emotions. Since then, the construct has been broadened and partially modified. Today this describes a condition characterized by an altered emotional awareness which leads to difficulties in recognizing your own and others' emotions. In half a century, the volume of scientific products focusing on alexithymia has exceeded 5,000. Such an expansive knowledge domain poses a difficulty for those willing to understand how alexithymia research has developed. Scientometrics embodies a solution to this issue, employing computational, and visual analytic methods to uncover meaningful patterns within large bibliographical corpora. In this study, we used the CiteSpace software to examine a corpus of 4,930 publications on alexithymia ranging from 1980 to 2020 and their 100,251 references included in Web of Science. Document co-citation analysis was performed to highlight pivotal publications and major research areas on alexithymia, whereas journal co-citation analysis was conducted to find the related editorial venues and disciplinary communities. The analyses suggest that the construct of alexithymia experienced a gradual thematic and disciplinary shift. Although the first conceptualization of alexithymia came from psychoanalysis and psychosomatics, empirical research was pushed by the operationalization of the construct formulated at the end of the ‘80s. Specifically, the development of the Toronto Alexithymia Scale, currently the most used self-report instrument, seems to have encouraged both the entrance of new disciplines in the study of alexithymia (i.e., cognitive science and neuroscience) and an implicit redefinition of its conceptual nucleus. Overall, we discuss opportunities and limitations in the application of this bottom-up approach, which highlights trends in alexithymia research that were previously identified only through a qualitative, theory-driven approach.

## Introduction

The word “Alexithymia” stems from old Greek and literally means “without words for emotions” [“a”=lack + “lèxis”=word + “thymos”=mood or emotion, see Lesser (1)]. This term describes a personality construct characterized by a deficit in emotional awareness and was firstly introduced at the beginning of the 70s by Peter Sifneos [cf. e.g., (2)] to denote the particular characteristics of people suffering from a variety of psychosomatic diseases (i.e., ulcerative colitis, asthma, peptic ulcer, rheumatoid arthritis). Indeed, the observations conducted on similar patients revealed that their physical symptoms were accompanied by a general inability to verbalize their emotions which prevented them from successfully engaging in a talking therapy. Even though the symptoms were related primarily to the patient's capacity to express their emotions through words, Sifneos et al. realized that the linguistic problem was only the surface of a deeper underlying issue. The main features of alexithymia they identified were chiefly: (i) difficulty in identifying feelings and distinguishing between feelings and bodily sensations of emotional arousal; (ii) difficulty in describing feelings to others; (iii) externally oriented thinking; and (iv) limited imaginal capacity (3).

In a short time, this psychological construct captured the attention of a number of researchers within the psychiatric community interested in the treatment of psychosomatic diseases. Indeed, in 1976, alexithymia was the main theme of the 11th European Conference on Psychosomatic Research (ECPR) held in Heidelberg, Germany (4). After a brief period in which alexithymia was hypothesized to identify a specific type of psychopathological personality with categorical characteristics (i.e., a nosological category) positively correlated with psychosomatic disorders, it became clear that alexithymia is instead a personality trait with dimensional and multifactorial characteristics which is shared also by other clinical populations (5). Alexithymia was also found to be normally distributed in the general population (6, 7), further confirming the continuous nature of the construct.

The search for a more precise definition of this construct was accompanied by the need to identify reliable instruments for its assessment. First of all, Sifneos developed the Beth Israel Hospital Questionnaire (BIQ; 2); shortly thereafter with a colleague, he put forward the Schalling-Sifneos Personality Scale (8, 9). At the beginning of the 1980s, Kleiger and Kinsman (10) proposed the MMPI Alexithymia Scale (MMPI-A) obtained by selecting a number of items related with BIQ scores from the Minnesota Multiphasic Personality Inventory. In the mid-80s, Krystal et al. (11) developed the Alexithymia Provoked Response Questionnaire (APRO) which was derived from a particular version of the BIQ. These questionnaires did not take into consideration the psychometric standard of test construction and provided only minimal empirical support for the construct of alexithymia.

A psychometrically sound measure of alexithymia started to be developed at the University of Toronto by the “Toronto group” in the mid-80s under the name of Toronto Alexithymia Scale [TAS; (12)]. This attained its final form as a self-report scale consisting of 20-items (TAS-20) about a decade later (13). Its validity has been tested in conjunction with other personality constructs and through several translation in other languages [cf. e.g., (14)]. TAS-20 also has the advantage of being brief and easy to administer. For these reasons, it is the most widely used measure for the assessment of alexithymia. This does not mean that the TAS-20 is the only psychometrically valid tool currently used for the assessment of alexithymia. Indeed, at least one alternative instrument must be mentioned, e.g., the Bermond-Vorst Alexithymia Questionnaire (BVAQ), a self-report measure which generally corresponds quite closely to dimensions of the TAS-20 (15).

The development of a standardized instrument allowed researchers to study alexithymia in relation to various clinical and non-clinical phenomena. This has made it possible to substantiate the observation that alexithymia is related with psychosomatic diseases. Moreover, alexithymic characteristics were found in various clinical conditions characterized by a disordered affect regulation (5), such as depression (16), self-harm and suicidality (17–19), schizophrenia (20), eating disorders (21, 22), substance use disorder (23) and autism spectrum disorder (24, 25). Hence, alexithymia started to be considered a non-specific vulnerability factor involved in the development of physical and mental disorders as well as a specifier associated with adverse outcomes when treating such conditions [see e.g., (26, 27)]. Concurrently, alexithymia was associated with several medical conditions such as diabetes, cancer or chronic illnesses (28–30).

Today, alexithymia is regarded as a personality trait and as a “sub-clinical phenomenon” (31) and it was never included in the Diagnostic and Statistical Manual of Mental Disorders (DSM), although it is recognized as one of the main clusters within the Diagnostic Criteria of Psychosomatic Research (32, 33). From a non-clinical perspective, the construct of alexithymia has drawn the attention of people interested in exploring wider psychological issues concerning emotional competence (34), emotional intelligence (35, 36) as well as related questions regarding empathy and theory of mind [cf. e.g., (37–41)].

As this introduction briefly illustrates, the history of the alexithymia construct is quite complex. Even though this is originally defined in a restricted domain, in time it has become more transversal and relevant for the study of a wide range of phenomena. In fact, today alexithymia appears to be essential to understand–among other things–how emotions and emotion regulation work. For this reason, the literature counts a dozen books and more than 5,000 scientific publications on alexithymia, retrievable from the main bibliographic databases (i.e., Web of Science, Scopus, Google Scholar, PsycInfo, PubMed). Many attempts have been made to analyze this literature in order to identify how the debate on this construct is articulated. According to the Web of Science database, there are more than 200 reviews issued on this topic from the beginning of the discussion on alexithymia till now. Some of these played a significant role in the development of this concept. Considering only the most recent times (from 2005 to 2020), 50 systematic reviews based on meta-analytical methods were published. These papers usually focus on specific aspects of alexithymia (i.e., on its relation with eating disorders, risky drinking, suicide, etc.) and they are often aimed at disambiguating the divergence in empirical results of single studies.

Despite previous literature providing a widespread overview of specific theoretical and empirical aspects of alexithymia, to our knowledge none of these works make use of a quantitative approach to investigate the complex and transversal character of this construct in its evolution and multiple ramifications. In fact, different from previous meta-analyses focusing on individual aspects of alexithymia, our study aims at approaching alexithymia research as a whole and analyzes how the scientific production on this condition is organized. Among other things, we would like to identify distinct areas in alexithymia research and explore how they change with time. We also aim to investigate which topics of interests are in the various development stages of the construct and which methods and techniques have been used to address alexithymia. In this way, we aim to reconstruct the same organic image of alexithymia research previously portrayed in theoretical and historical literature reviews, but through a bottom-up approach borrowed from Scientometrics.

Scientometrics applies quantitative methods to measure and shape the development of science, seen as an informational process (42). Some of the main themes in Scientometrics include ways of measuring research quality and impact, understanding the processes of citations, and mapping scientific fields and the use of indicators in research policy and management (43). For our purposes, Scientometrics can help us to deal with the difficulties arising from the exponential growth of the literature on alexithymia in the last few decades by providing a means to organize it in meaningful structures [for a review of the utility of Scientometrics for this purpose, see e.g., (44, 45)].

The bottom-up approach used in scientometric reviews has already been proved successful to assess trends and the evolution of assessment instruments or techniques developed in narrow disciplinary domains such as linguistics and psychology (46, 47).

In the present study, (i) a corpus of scientific documents is identified via the Web of Science (WoS) search algorithms; (ii) the identified articles and their references are segmented and classified by co-citation techniques developed by CiteSpace software (48–50); and (iii) they are analyzed in their content, according to the prominent trends identified by the CiteSpace software.

Within Citespace, cited papers are represented by interactive maps across the domain of space and time to obtain a depiction of almost 50 years of research in alexithymia across the world. The algorithms developed to measure the strength of links between citing and cited publications are able to create clusters of papers or journals focusing on thematically distinct aspects of alexithymia. In addition, reliable parameters implemented in the software can estimate the impact of publications or editorial venues on their specific cluster and on the overall corpus of documents retrieved on the topic. This allows us to identify the most influential publications on alexithymia and the most pursued issues considered by the research in relation to different time frames. Moreover, we can pinpoint the shifts in the research lines on this subject as well as of the approaches employed to investigate this condition.

To summarize, this study examines the extent to which the historical reconstructions of alexithymia research suggested in traditional narrative reviews are replicable using a bottom-up scientometric approach based on knowledge mapping techniques.

## Materials and Methods

### Data Source and Descriptive Statistic

**Figure 1:**
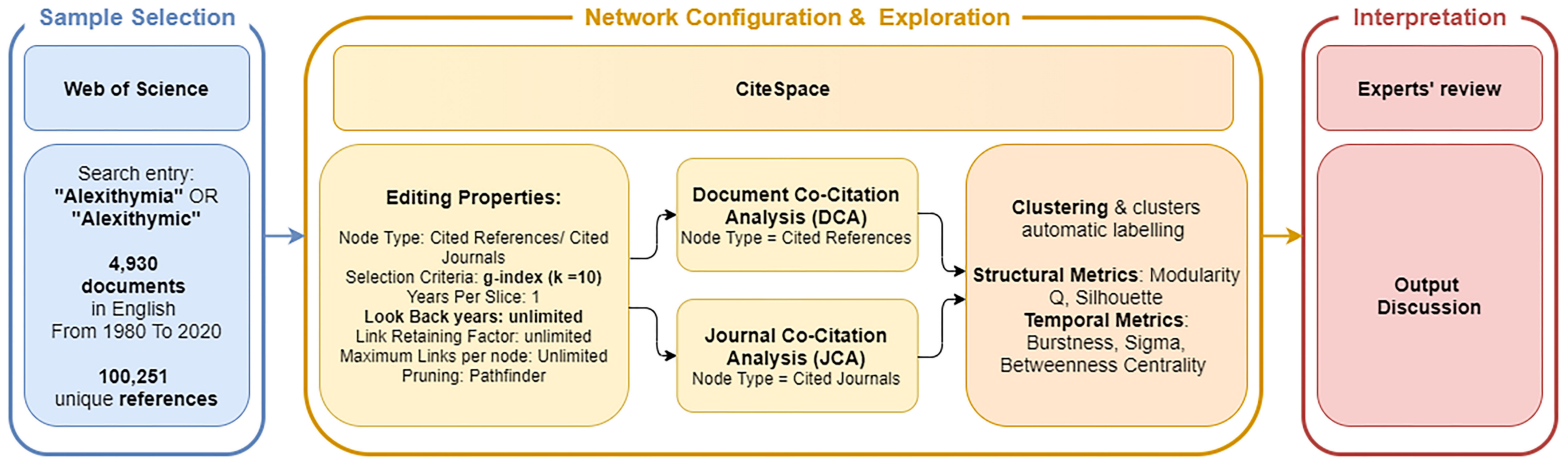
Overview of the study protocol.

The data used for the analyses include 4,930 publications on the conceptual analysis, assessment and neurophysiological correlates of Alexithymia published between January 1st 1980 and May 1st 2020, with 100,251 references downloaded from the WoS Core Collection. The reason for using WoS is due to its coverage of published research on alexithymia, which is higher than alternative databases such as Scopus, Pubmed or EBSCO. The search syntax adopted for this study was Topic (“Alexithymia”) OR Topic (“Alexithymic”). By means of the search field Topic, WoS extracts title, abstract, and keywords. From an initial pool of 5,390 documents, we excluded those that were written in languages other than English, thus arriving at a final sample of 4,930 publications with their 100,251 references (see Figure 1).

Supplementary Figure 1 reports the number of documents by year. Overall, it can be observed that there was an exponential growth in the research on Alexithymia from its start till the present day. The WoS database contains <10 publications per year dating from the early 80s; these reached a median of ~100 publications per year for the first time in the year 2002 and then a median of 200 in 2010. They joined their highest point in 2019 with more than 400 publications, which alone corresponded to 9% of the overall production on alexithymia stored in the WoS database.

Supplementary Table 1 presents the results of frequency analysis performed on the sample of 4,930 publications which revealed document typologies and the most productive authors, universities/institutes, and countries/regions. Considering the document typologies defined by WoS, most of the documents fell under the category “Articles” (78%), which was followed by the category “meeting abstract” (13%), “review” (3.7%) and other few categories with lower incidence. Among the most prolific authors, there were the three authors (G. J. Taylor, R. M Bagby and J. D. A Parker) of the Toronto Alexithymia Scale (TAS-20), respectively, at first, second and fourth position (with 91, 83, and 62 publications). Other prolific authors were O. Luminet (70 publications), Fukunishi (56 publications), and M. Youkamaa (55 publications). As for the most prolific organizations, the University of Toronto topped the list with 148 publications, followed by the University of London with 99 publications and the University of Rome “La Sapienza” with 95 publications. Among the most prolific countries, USA topped the list with 1,155 publications, contributing 23% of the overall production. It followed Italy (16%), England (9%), and Canada (8%). The first two Asian countries were Turkey (3.5%) and Japan (3.2%), respectively, at the 11th and 12th position.

### Data Analysis and Visualization With CiteSpace

The data were exported from the WoS, producing text files containing the “full record and cited references” for each of the 4,930 publications retrieved. CiteSpace software, Version 5.6.R5 (48–50), was employed in order to analyze the data. Document co-citation analyses (DCA), which represents the number of times two publications were co-cited (cited together) in later publications, was employed to individuate time-evolution of the most influential publications and the most pursued themes and techniques, while journal co-citation analysis (JCA), which represents the number of times two journals were co-cited (cited jointly) in publications, was adopted to differentiate the time evolution of disciplinary domains. It should be noted that a small amount of references (0.3%) could not be processed by the software during co-citation analyses. We consider this percentage as a negligible loss of data (51).

CiteSpace was used to generate and analyze two types of networks of co-cited references via DCA and JCA. The DCA and JCA networks were computed separately, using the same criteria described below. The time span of the two networks ranged from 1980 to 2020, with the time slicing configuration at 1 per year. We compared three criteria for node selection to identify the optimal DCA and JCA networks: Top N, Top N%, and g-index. Top N function picks up the N most cited articles and uses information from them to form the network for each time slice. Similarly, Top N% includes the Top N% most cited articles in each time slice to construct the network. G-index criterion (52) represents a variant of h-index, which considers the number of citations of an author's most important publications. Specifically, the g-index is the “largest number that equals the average number of citations of the most highly cited g publications” (51). The networks built with Top N with N at 50, Top N% with N at 10, and g-index with a scaling factor at 10 and 25 were compared. Eventually, we selected g-index criterion with a k scaling factor of 10 because it displayed greater silhouette and modularity indexes and a major consistency in cluster structure. Moreover, the cluster configuration could be better replicated using different versions of the software. In the construction of the network, we edited properties in a way to be as comprehensive as possible. Therefore, the “Look back years” parameter was set at −1, indicating that all the references cited in a citing paper were considered to construct the network, independently from their temporal distance from the source paper. At the same time, we set the “Link Retaining Factor” and the “Maximum Links per node” parameters as unlimited, thus allowing the program to explore all the links between cited publications. After a first visualization of the network, we decided to apply the Pathfinder function. This function consists of a link reduction algorithm (53), which gave us a more predictable and interpretable network configuration.

#### Metrics of Interest

Modularity Q index and average silhouette metrics were considered to detect the overall structure of the networks, while burstness, betweenness and sigma metrics were considered to assess how the structural and temporal properties of single nodes (publications, journals) impact the networks.

##### Modularity Q Index

A network's modularity is a global measure of the overall structure of the network (54, 55), which measures the extent to which a network can be decomposed into multiple components, or modules. The modularity Q index ranges from 0 to 1, where, as a rule of thumb, a value close to 1 means that the network is clearly divided into distinct groups.

##### Silhouette

The silhouette value of a cluster measures the quality of a clustering configuration. Its value ranges between −1 and 1, where values over 0 indicate major homogeneity (50). A silhouette value can be computed for the overall network or for the inner clusters. It might be considered that small clusters usually present a stronger silhouette due to the higher homogeneity of small samples.

##### Burstness

Citation burstness measures the burst of citations to a given node. CiteSpace explores the bursts of nodes within a given network through Kleinberg's algorithm (56). Burstness index can be detected for single nodes (references, journals etc.) or for entire clusters. If a cluster contains numerous nodes with strong citation bursts, then, the cluster as a whole captures an active area of research or an emerging trend.

##### Betweenness Centrality

Betweenness centrality (57, 58) measures the extent to which paths in the network go through a certain node. In CiteSpace, betweenness centrality scores are normalized to the unit interval [0, 1]. A node of high betweenness centrality is usually one that connects two or more large groups of nodes with the node itself as the in-between, hence the term betweenness. A node with a strong betweenness centrality score represents a paper or a journal with great influence inside the network. CiteSpace highlights nodes with high betweenness centrality with purple trims. The thickness of a purple betweenness centrality trim is proportionate to the strength of its betweenness centrality.

##### Sigma (∑)

A composite metric sigma is defined in CiteSpace to measure the combined strength of structural and temporal properties of a node, namely, its betweenness centrality and citation burst. Sigma is computed as (centrality +1)^burstness^ (50), with higher values indicating works with higher influential potential.

#### Cluster Visualization and Labeling

**Figure 2:**
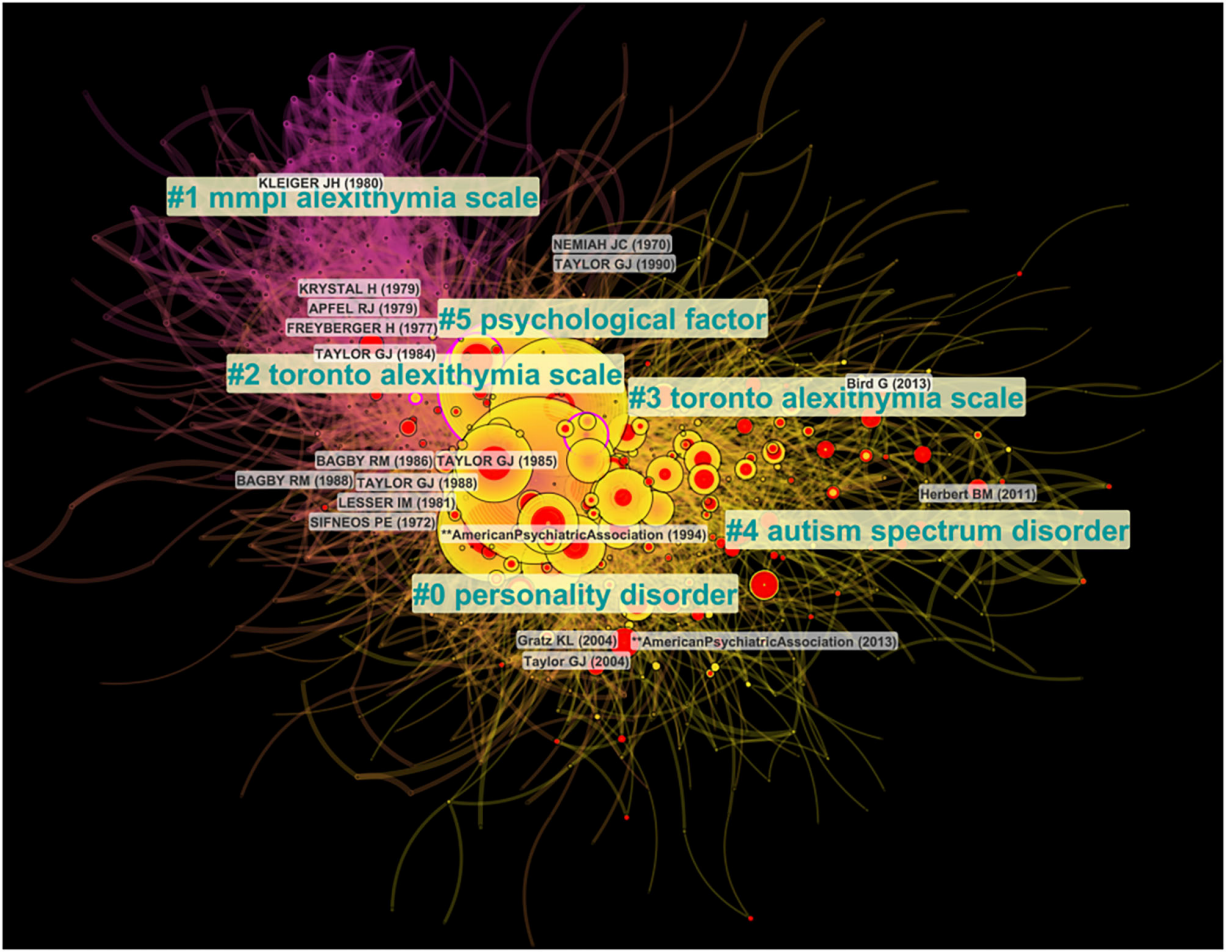
Cluster view of the document co-citation analysis (DCA) generated using CiteSpace Version 5.6.R5 (48–50). Modularity *Q* = 0.51; average silhouette = 0.75. Colored shades indicate the passage of the time, from past (purplish) to the present time (reddish). Colored tree rings refer to the nodes with high betweenness centrality (purple tree rings) and burstness (red tree rings).

**Figure 3:**
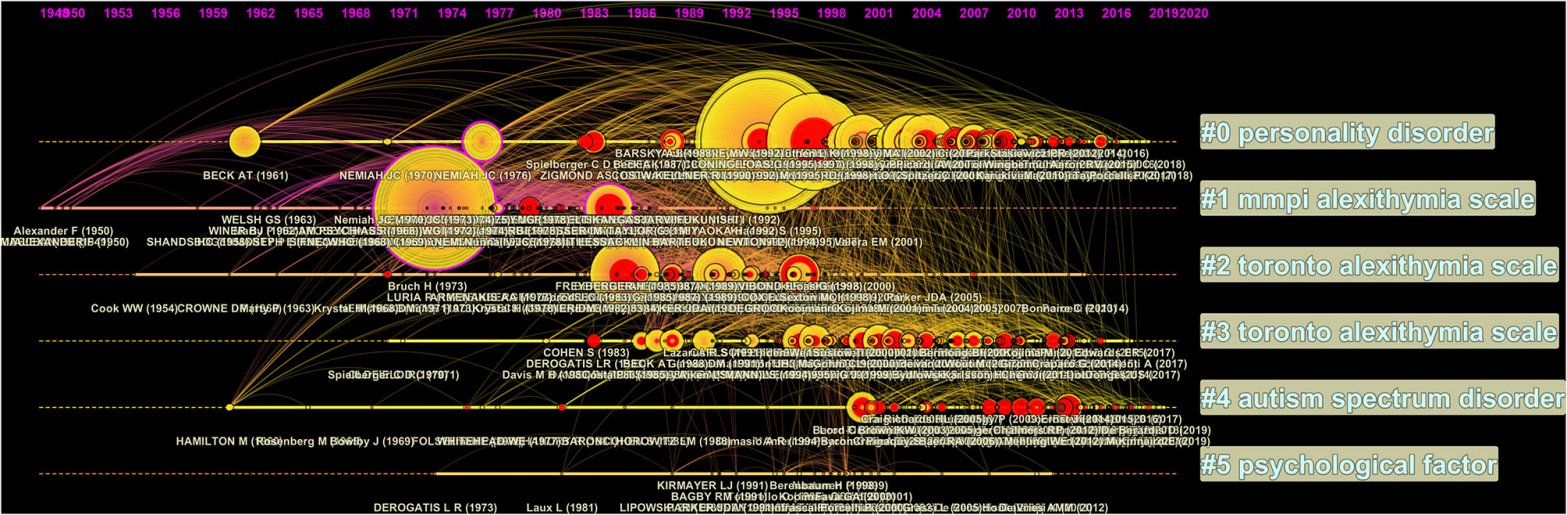
Timeline view of the document co-citation analysis (DCA) generated using CiteSpace Version 5.6.R5 (48–50). Modularity Q = 0.51; average silhouette = 0.75.

The clustering function in CiteSpace has been used to find the major entities in which single nodes could be grouped inside the network. Clusters are numbered depending on their size, starting with the largest (#0) to the smallest (#5). In CiteSpace, version 5.6.R5, three functions are available to label clusters: Log-Likelihood Ratio (LLR), Latent Semantic Indexing (LSI) and Mutual Information (MI). The three methods have been applied to the current DCA networks. We implemented the LLR function, given the higher informative content associated with the output labels. This approach is supported by the same software creator, who pointed out the best performance of LLR in terms of “uniqueness and coverage” of labels (59). Therefore, we used two visualization methods to analyze our networks: the cluster view and the timeline view. The cluster view (Figure 2) produces a spatial representation of the network shaped over time, where the layout of clusters inside the network reflects the connections between their nodes. The size of the circles reflects the amount of cited references inside the clusters. The colored shades indicate the passage of the time, from past (purplish) to the present time (reddish)–that is, from cold colors to warm colors. For instance, since Cluster #1 has many bluish and purple edges and nodes, we can infer that it is the oldest cluster among all. On the other hand, colored tree rings refer to the nodes with high betweenness centrality (purple tree rings) and burstness (red tree rings). In the timeline view (Figure 3), major clusters are horizontally arranged. The earliest nodes are placed at the leftmost position, whereas the most recent ones are placed at the rightmost positions. Vertical links between nodes indicate citation links between publications belonging to different clusters.

## Results

### Document Co-citation Analysis (DCA)

The document co-citation analysis produced a network with modularity Q index and average silhouette metric of 0.51 and 0.75, respectively, suggesting moderate modularity and high homogeneity (Figures 1, 2).

Table 1 shows the six major research clusters detected using the clustering function and labeled using the LLR function. Clusters were numbered in descending order based on the cluster size, starting from the largest cluster #0 (“Personality Disorder” size = 185; silhouette = 0.64; mean year = 2002), to the smallest #5 (“Psychological factor”; size = 27; silhouette = 0.93; mean year = 1997). With regard to the time span covered by clusters, it should be noted that overall, all clusters were very extensive in the time domain. Indeed, cluster duration ranged from 39 to 60 years, thus presenting frequent overlaps in time. This result was consistent with the edit properties selected, with the “Look back year” parameter set as unlimited. Nevertheless, it was still possible to distinguish cluster timelines on the basis of the mean year for each cluster and the visualization of major bursts within each cluster (see Figures 2, 3). For instance, cluster #1 (“MMPI alexithymia scale”) was the oldest one (mean year = 1978), as understandable also by its purplish color (Figure 2). On the contrary, cluster #4 (“Autism spectrum disorder”) represented the most recent one (mean year = 2005), as understandable by its light yellowish color in Figure 2. Relying on the timeline visualization (Figure 3) and on the mean years, we could distinguish clusters #2 and #3, both titled “Toronto Alexithymia Scale” by the Log-Likelihood Ratio (LLR) function. Within CiteSpace, cluster labels are chosen with reference to the title terms, keywords, and abstract terms of citing documents (49). Therefore, the term “Toronto Alexithymia Scale” could have been mentioned by several influential citing publications in these two clusters, and thus it was chosen by the LLR function. However, cluster #2 (mean year = 1990) is chronologically anterior to cluster#3 (mean year = 2002). Concerning the biggest cluster #0 (“Personality Disorder”), we noticed a sustained activity with bursts occurring at spots distant in time (see Figure 3). This result suggests a higher heterogeneity within this cluster, as confirmed by the moderately low silhouette value (= 0.64) and by the analysis of its most representative documents (see the section Discussion).

**Table 1:** Clusters computed via Document co-citation Analysis (DCA).

**Cluster ID**	**Cluster label**	**Size**	**Silhouette**	**Mean (year)**	**From**	**To**	**Duration of the cluster (in years)**
0	Personality Disorder	185	0.64	2002	1961	2018	57
1	MMPI alexithymia scale	147	0.86	1978	1948	2001	53
2	Toronto alexithymia scale	147	0.62	1990	1954	2014	60
3	Toronto alexithymia scale	140	0.71	2002	1970	2017	47
4	Autism spectrum disorder	73	0.82	2005	1960	2019	59
5	Psychological factor	27	0.93	1997	1973	2012	39

*Cluster labels are obtained via the LLR method. Cluster IDs are in decreasing order depending on cluster size. Size equals the number of cited documents within the cluster. Cluster duration in years depends on the time span from the oldest to the most recent paper. Silhouette is an index of cluster homogeneity, with values approaching 1 indicating the maximum in homogeneity*.

To understand what the most active areas in the network are, the document bursts have been computed via document co-citation analysis. Among the initial pool of publications and their references, citation bursts sustained for at least 2 years were found for 407 references. In addition, in order to detect which documents had a strategic role in connecting more clusters, we computed betweenness centrality, while publications with potential scientific novelty were explored through the metric sigma.

Table 2 shows the top 25 documents in terms of burst strength, with repetition to the time span of each citation burst (for a complete list, please see Supplementary Table 2). Among these, 10 publications belonged to Taylor GY, Bagby RM, and Parker JDA, the group of researchers known for having developed the Toronto Alexithymia Scale (TAS). The publication attesting to the development of the original TAS (12) was in the first position for burst strength (=70.11). Within Table 2, many were early publications playing a crucial function for a conceptual definition of alexithymia (1, 8, 10, 67, 68, 72, 78). Consequently, these were also the publications with the earliest burst beginnings (between 1981 and 1985) and the longest burst duration (lasting between 15 and 21 years). Conversely, the most recent publications within the list (24, 71, 75) presented the shortest burst duration (between 3 and 5 years). However, for these papers, the increasing trend in citation (the burst) was also found in 2020, suggesting that their burst strength and, therefore, their influence is likely to continue for the foreseeable future. Among the other documents appearing in Table 2, there were also two different versions of the Diagnostic and Statistical Manual of Mental Disorders (DSM-IV, DSM-V), suggesting alexithymia has widely been explored in conjunction with other clinical conditions. Then, we noted that among the documents with highest burst, there were the two influential books by Taylor et al. (5) and Krystal and Krystal (76).

**Table 2:** List of the top 25 documents for burst strength, estimated via document co-citation analysis (DCA).

**References**	**Publication title**	**Burst strength**	**Burst begin**	**Burst end**	**Centrality**	**Sigma**	**Cluster ID**
Taylor et al. (12)	Toward the development of a new self-report alexithymia scale.	70.11	1988	2003	0.07	151.25	2
Taylor (60)	Alexithymia: concept, measurement, and implications for treatment.	64.39	1985	2003	0.11	692.91	1
American Psychiatric Association (61)	American Psychiatric Association. DSM-V	58.38	2015	2020	0.01	1.71	4
Apfel and Sifneos (8)	Alexithymia: Concept and measurement.	57.74	1981	2001	0.07	38.48	1
Taylor et al. (62)	Criterion validity of the Toronto Alexithymia Scale.	54.25	1989	1998	0.03	5.94	2
Bagby et al. (63)	Toronto Alexithymia Scale: Relationship with personality and psychopathology measures.	49.15	1988	2002	0.03	3.41	2
American Psychiatric Association (64)	American Psychiatric Association. DSM-IV	39.53	1998	2012	0.05	6.89	0
Bagby et al. (65)	Alexithymia: a comparative study of three self-report measures.	38.07	1988	2001	0.01	1.43	2
Gratz and Roemer (66)	Multidimensional assessment of emotion regulation and dysregulation	37.43	2016	2020	0.03	2.58	3
Krystal (67)	Alexithymia and psychotherapy	37.35	1981	2001	0.06	7.81	1
Kleiger and Kinsman (10)	The development of an MMPI alexithymia scale.	36.89	1981	1998	0.04	3.98	1
Freyberger (68)	Supportive psychotherapeutic techniques in primary and secondary alexithymia.	36.20	1982	2003	0.13	76.13	1
Lesser (1)	A review of the alexithymia concept.	35.79	1983	1998	0.05	4.89	1
Sifneos (2)	Short-term psychotherapy and emotional crisis.	33.68	1981	2001	0.06	7.53	1
Taylor et al. (69)	Validation of the alexithymia construct: A measurement-based approach.	33.59	1992	2000	0.01	1.24	2
Taylor and Bagby (70)	New trends in alexithymia research.	32.31	2006	2012	0.04	3.56	0
Herbert et al. (71)	On the relationship between interoceptive awareness and alexithymia.	31.90	2015	2020	0.01	1.22	4
Bird and Cook (24)	Mixed emotions: the contribution of alexithymia to the emotional symptoms of autism.	31.90	2015	2020	0.02	1.93	4
Nemiah and Sifneos (72)	Affect and fantasy in patients with psychosomatic disorders	31.54	1985	2006	0.04	3.77	2
Parker et al. (73)	Factorial validity of the 20-item Toronto Alexithymia Scale.	30.40	1995	2006	0.03	2.23	2
Taylor et al. (5)	Disorders of Affect Regulation. Alexithymia in Medical and Psychiatric Illness.	29.98	1998	2010	0.03	2.57	0
Taylor et al. (74)	Alexithymia and somatic complaints in psychiatric out-patients.	29.47	1994	2002	0.03	2.15	2
Bird et al. (75)	Empathic brain responses in insula are modulated by levels of alexithymia but not autism.	28.67	2013	2020	0.01	1.53	4
Krystal and Krystal (76)	Integration and Self-Healing: Affect, trauma and alexithymia	28.27	1989	2001	0.03	2.48	2
Blanchard et al. (77)	Psychometric properties of a scale to measure alexithymia.	27.82	1982	1992	0.02	1.68	1

*For each document are reported burst begin and end, betweenness centrality, sigma and the Cluster ID to which they belong*.

Analyzing the documents with highest sigma values (see Supplementary Table 3), we could notice that documents at the top of the list enormously outpaced all the others. This is consistent with the measurement and the conceptualization of sigma as an index of scientific novelty and with case studies by Chen et al. (79) showing that highest sigma values were usually associated with Nobel Prize and other award-winning researchers. On the other hand, overall the publications within our network had a low betweenness centrality index ranging from 0.01 to 0.14 (see Supplementary Table 3). The highest centrality score (=0.14) belonged to Sifneos (2) who introduced the term “alexithymia” for the first time, while the highest sigma value (=693.31) belonged to the review of the state of the art research in alexithymia by Taylor (60). The influence of this last publication is also attested by the fact that this was the second record for burst strength and the third record for betweenness centrality (0.11). Other influential documents were the paper presenting the TAS (12), first for burst strength and second for sigma value (=151.25), and the paper where Freyberger (68) introduced the concept of “secondary alexithymia,” which is attested third for sigma (=76.13) and second for centrality score (=0.13).

### Journal Co-citation Analysis (JCA)

The JCA produced a network with a total modularity Q score at 0.47 and an average silhouette score of 0.74, indicating moderate modularity and high homogeneity. JCA represents an aggregation at a higher level than DCA, since articles published in the same journal are lumped together to form a supernode in a network of journals. Inside the JCA network, 332 nodes (journals) had a burst history of at least 2 years.

Table 3 shows sample journal bursts computed via Journal Co-citation Analysis (JCA). It should be noted that CiteSpace was not able to capture the difference between book titles and journals automatically. Therefore, in displaying results (Table 3) we selected only journals (for a complete list, please see Supplementary Table 4).

**Table 3:** List of the top journals for burst strength, estimated via Journal Co-citation Analysis (JCA).

**Journal**	**Strength**	**Begin**	**End**	**Duration**
Psychotherapy and Psychosomatics	164.51	1980	2006	26
Psychosomatic Medicine	138.78	1980	2006	26
American Journal of Psychiatry	125.12	1985	2006	21
Frontiers in Psychology	88.00	2016	2020	4
Plos One	74.82	2015	2020	5
Modern Trends in Psychosomatic Medicine	73.19	1980	2001	21
Journal of Nervous Mental Disease	65.90	1981	2004	23
American Journal of Psychotherapy	58.20	1981	2002	21
Psychosomatics	56.71	1992	2005	13
Short Term Psychotherapy	51.76	1981	2001	20
British Journal of Psychiatry	47.05	1993	2006	13
Psychiatric Clinics of North America	46.64	1988	2001	13
Psychological Reports	46.48	1995	2007	12
British Journal of Medical Psychology	40.12	1990	2007	17
New England Journal of Medicine	35.91	1985	2008	23
General Hospital Psychiatry	35.26	1981	2001	20
Int J Psychoanalytic Psychotherapy	34.19	1984	2004	20
European Journal of Personality	33.69	1995	2008	13
New Trends Exp. Clin. Psychiat	31.10	1995	2003	8
Frontiers in Psychiatry	31.05	2017	2020	3
Soc Cogn Affect Neur	29.04	2014	2020	6
Scientific Reports-Uk	29.03	2017	2020	3
Journal of Human Stress	28.82	1986	2004	18
Frontiers in Human Neuroscience	27.74	2015	2020	5
Neuroscience & Biobehavioral Reviews	27.12	2016	2020	4

The journals in the first two positions for burst strength were, respectively, *Psychotherapy and Psychosomatics* (strength = 164.51) and “Psychosomatic Medicine.” These journals stand out also for the longest duration of their citation burst, estimated at 26 years. The citation burst for these journals dated back to 1980, but it could be more distant in reality, considering the time limitation of the WoS database, which collected documents starting from 1980. Other journals whose citation burst started in the early ‘80s report all in their titles the terms “psychosomatics” and “psychotherapy” (*Modern Trends Psychotherapy, American Journal of Psychotherapy, Short Term Psychotherapy, and Modern Trends in Psychosomatic Medicine*). From DCA, we observed that the papers with the highest citation burst, centrality and sigma (Table 2) published during the ‘70s fell within the aforementioned journals [see (57, 58, 59, 8, 60]. Additionally, the journals within the psychiatric discipline presented a long duration in citation burst (between 13 and 23 years). Among these, the *American Journal of Psychiatry* stands out as the most influential one (third for burst strength = 125.12). Other journals within the psychiatric discipline were the *Journal of Nervous Mental Disorders*, the *British Journal of Psychiatry, Psychiatric Clinics of North America* and the *General Hospital Psychiatry*. Influential journals with still active citation bursts were *Frontiers in Psychology* and *Plos One*, which were fourth and fifth ranked for burst strength respectively, despite the limited burst duration (4-5 years). Lower in the list, there were *Frontiers in Psychiatry, Social Cognitive and Affective Neuroscience, Scientific Reports—UK, Frontiers in Human Neuroscience*, and *Neuroscience & Biobehavioral Reviews*, all active between 2015 and 2020.

## Discussion

### Major Thematic Clusters Found via DCA

The content of major clusters obtained via document co-citation analysis (DCA) is discussed below. Cluster presentation follows a chronological order (for a list of the main citing and cited publications subdivided by cluster ID, please see also Supplementary Tables 5, 6).

#### Cluster #1: “MMPI Alexithymia Scale”

Cluster #1 is the oldest and it contains some of the major papers written between the ‘70s and the early ‘80s, which significantly contributed to the definition of the construct and to research on its etiology. The title of the cluster (“MMPI Alexithymia Scale”) refers to one of the original assessment instruments, developed by Kleiger and Kinsman (10). This is due to the fact that many influential citing documents within cluster #1 refer to the MMPI Alexithymia Scale in their title, abstract or keywords (see Supplementary Table 5). Although this title is not representative of the topic of the cluster, it captures the fact that the most influential papers date back to a period preceding the development of TAS. Indeed, major bursts refer to studies which presented or validated the first instruments to measure the construct of alexithymia: the Beth Israel Hospital Psychosomatic Questionnaire (2), the Schalling-Sifneos Personality Scale (8), and the already mentioned 22-item MMPI Alexithymia Scale (10). Among the other influential publications included in this cluster, there are the seminal publications by Sifneos on the construct of “alexithymia” (2, 78). A pivotal role is also played by Freyberger's (68) paper, introducing the concept of “secondary alexithymia”: i.e., a condition acquired in the adulthood as a consequence of an organic disease, a chronic illness or an invasive medical treatment (i.e., dialysis, transplant). The contribution of Krystal in defining the etiology of the construct is evident in two prominent publications on patients with substance abuse behaviors and clinical cases of post-traumatic stress disorder (PTSD) (67, 80). The paper with the highest burst strength and sigma value within the cluster and the overall network is a review by Taylor (60), which summarizes the knowledge on alexithymia collected until that time, illustrating the medical and psychiatric disorders that are usually associated with it as well as the diagnostic instruments developed so-far and the difficulties to treat, with psychotherapy, people suffering from alexithymia. This publication probably represented a turning-point since it provided a systematic framework for the theoretical foundations of the construct and, at the same time, it highlighted the limitations of the knowledge available at that time, preparing the field for the development of a new assessment instrument, presented by Taylor et al. just 1 year later (12).

In brief, Cluster #1 collects the early literature on alexithymia that goes from the '70s to the mid-1980s, a period which is antecedent to the advent of the Toronto Alexithymia Scale and was focused on the theoretical definition of the construct mainly in connection with psychosomatic diseases.

#### Cluster #2: “Toronto Alexithymia Scale”

The title of this cluster refers to the Toronto Alexithymia Scale [TAS, (12)] and it appears appropriate for this group of articles. The scale discussed by the papers included in this collection is the first version of the TAS. The articles based on the final version of the Toronto Alexithymia Scale (TAS-20) are included in cluster #0, named “Personality Disorder.” Therefore, this cluster represents the first stage of the research program fostered by the researchers of the University of Toronto, which aimed to evaluate the validity of the construct of alexithymia “using a measurement-based, construct validation approach” (69). Within this cluster, major bursts are all represented by papers written by one or more exponents from the group of Toronto. Two are the exceptions to this intellectual monopoly. A first one is represented by Krystal's book (76) which provides an overview on the link among alexithymia, emotional regulation and traumatic experiences and which drives the attention of the research on an aspect of alexithymia which was mostly neglected, i.e., its relationship with anhedonia. Indeed, people who suffer from an altered emotion awareness also exhibit an altered sensibility of the hedonic tones of their experience, especially of the positive ones. The second exception is surprisingly represented by an earlier publication by Nemiah and Sifneos (72), which presented excerpts of clinical interviews with patients suffering from psychosomatic disorders as well as some notes by the authors on them. The patients' reports included in this article describe the characteristics that 3 years later will be considered as crucial to qualify the alexithymic condition. The relevance of this paper became clear only after the work of the Toronto Group and their efforts to identify the main factors characterizing the construct of alexithymia, as witnessed by the late burst beginning, dating back to 1985 (see Table 2). All other prominent publications within the cluster discuss the psychometric properties of the first version of the Toronto Alexithymia Scale: they present its criterion validity (62) or test its construct validity (63) or, again, they compare this instrument with previous ones such as the Schalling-Sifneos and the MMPI-A scales (65). Of prominent relevance is also the subsequent review by Taylor et al. (81), first for centrality (=0.09) within the cluster. By suggesting that alexithymia represents “a potential new paradigm for understanding the influence of emotions and personality in physical illness and health,” this paper paves the way for a conceptualization of alexithymia as a non-specific vulnerability factor that can potentially represent a predisposition for the onset of personality disorders and/or of disorders related with emotional dysregulation. Indeed, within the clusters some influential papers focused on the use of TAS to assess alexithymic characteristics in new clinical conditions such as feeding and eating disorders (82–84). Another line of research focuses on the relationship between depressive and anxiety disorders and alexithymia, as measured with TAS (85, 86).

To sum up, the cited publications within cluster #2 cover the period between 1985 and the early ‘90s. They include studies which are mainly related to the first version of the Toronto Alexithymia Scale. In the clinical contexts, these studies focused primarily on the relationship between alexithymia and eating disorders, anxiety or depression.

#### Cluster #0: “Personality Disorder”

This cluster, with its 185 cited references, is the largest one. Here, the most influential publications start from 1994, in other words, after the development of the new 20-item Toronto Alexithymia Scale. Notably, the two publications presenting the psychometric properties of the new TAS-20 (13, 87) share an impressively high citation frequency (=1941; =1161), although they have no bursts or very low burst. This phenomenon could be due to the fact that the new scale did not represent a revolutionary change in the way alexithymia was assessed, contrarily from the first TAS, which ranked first position for burst strength and sigma. Therefore, these publications did not experience an abrupt explosion in citations, although they might have been cited by all the following studies employing this new version of TAS. Consequently, they represent the document with the highest number of citations in the overall DCA network. The central role of the “Toronto Group” in this cluster is also evident from other documents in first positions within the cluster for burst and sigma metrics, mainly: the book by Taylor et al. (5) and a subsequent review (88) exploring the relationship between alexithymia and, more generally, emotion regulation/dysregulation for mental and physical health in general; two methodological papers (89, 90) testing the validity and reliability of TAS-20 in different linguistic and cultural contexts; a review by Taylor and Bagby (70), which suggests a shift in research on alexithymia from “measurement-based validation studies to experimental investigations,” exploring the relationship between alexithymia and various aspects of emotional processing through the employment of standardized emotional task and the measurement of brain activity and physiological responses (see cluster #3, renamed by us “Emotion Information Processing”); a publication by Bagby et al. (91) presenting the Toronto Structured Interview for Alexithymia (TSIA), to be used together with the self-report TAS-20 to guarantee a multimethod assessment of alexithymia. Although there is certain heterogeneity in the cluster (silhouette = 0.64), influential publications by other authors can be thematically and methodologically connected with the lines of research pursued by the mentioned publications by the Toronto Group. For instance, the link between the ability to regulate emotion and the consequences for physical and mental health is captured in publications by Lumley et al. (92) and Grabe et al. (93). Moreover, few papers review specifically the relationship between alexithymia and depression (94) or alexithymia and alcohol abuse disorders (95). This fact explains why the DSM-IV (64) was the document with the highest citation burst within the cluster. Some publications continue to explore the psychometric properties of TAS in different populations (96) or in conjunction with other phenomena, such as the construct of personality (97) or the theory of the levels of emotional awareness (98). A paper by Ogrodniczuk et al. (99) reviews the challenges and the solutions developed to treat patients with alexithymia through psychotherapy. Finally, in a relatively high position for burst strength, there is a review by Sifneos (100), titled “Alexithymia: Past and Present” which is one of the most recent papers written by Sifneos on alexithymia.

In short, this cluster covers research on alexithymia spanning from the mid-1990s to the late 2000s. This period follows the development of the TAS-20 and it is largely influenced by the research of the Toronto Group. Articles by other groups employ this new self-report instrument to investigate the relationship that alexithymia entertains with a variety of clinical conditions or emerging constructs related to personality and emotional competence.

#### Cluster #5: “Psychological factor”

This cluster is relatively small, comprising exactly 27 cited papers. The majority of cited publications are contemporary to a number of those included in Cluster #0 (“Personality Disorder”) and in Cluster #3 (“Emotion Information Processing”), dating back to the second half of the ‘90s. Thematically, publications within this cluster address alexithymia as a risk factor for mental and physical health conditions. The relationship between alexithymia and depression is explored in a paper by Honkalampi et al. (101), first for citation burst within the cluster, and in later publications (102, 103), which examine the potential relationship between alexithymia and the regulation of the immune-inflammatory responses. Other works explore the relationship between alexithymia and defense style (104) or neuroticism (105). The influence of alexithymia for physical health is studied in conditions such as functional gastrointestinal disorders (106, 107), inflammatory bowel disease (108) or hypertension (109). More generally, this relationship is highlighted by bursts in publications referring to psychosomatic research (32, 110). Within the cluster, few influential publications still focus on the exploration of the psychometric properties of instruments prior to TAS-20. Among these, one study suggests a revision of TAS (111), while two publications by the Toronto Group question the values of previous instruments such as MMPI-A and SSPS-R (112, 113).

Overall, cluster #5 collects a small number of publications, posterior to the TAS-20, whose primary aim is to explore alexithymia in psychopathology and physical illness.

#### Cluster #3: “Toronto Alexithymia Scale” (Renamed: “Emotion Information Processing”)

The major publications within this cluster chronologically follow the development of TAS and TAS-20, while they precede chronologically the papers included in cluster # 4 (“Autism Spectrum Disorder”). The title automatically extracted by the LLR method does not capture the main topic of the cluster, although it captures the fact that the majority of empirical studies mentioned here employ the TAS-20 in their design. In accordance with Aryadoust et al. (114), highlighting the opportunity to employ cluster counter-labels based on expert evaluations of the content of documents within the cluster, we argue that an analysis of the major bursts suggests that a more appropriate title for this cluster would be “Emotion Information Processing.” Indeed, the most cited paper within the cluster is Lane et al. (115): this paper puts forward the hypothesis that alexithymia is not simply a problem of emotion labeling, but it is “associated with impaired verbal and non-verbal recognition of emotion stimuli and that the hallmark of alexithymia, a difficulty in putting emotion into words, may be a marker of a more general impairment in the capacity for emotion information processing” (115). Consistently with this idea, the most pursued line of research consists in the exploration of deficits in the recognition of facial expressions among individuals with alexithymia (116–119). Other studies in this cluster use a wide range of emotional tasks, testing the processing of emotionally salient pictures (120) or the processing of emotional prosody and semantics (121), or again the ability to match emotional stimuli of different nature (115). Some studies test the relationship between alexithymia and empathy (38, 122), mentalization (37) and emotional intelligence (123). As it is pointed out by meta-analyses and reviews on the neural correlates of alexithymia published after 2010 [cf. (124, 125)], the majority of these studies explore their hypotheses by applying neuroimaging techniques. Central within the cluster are the publications by Lane and Schwartz who at the end of the ‘80s put forward a cognitive-developmental theory of emotional awareness. They considered emotional awareness as the result of a cognitive processing which undergoes five levels of structural transformation of emotional information. They also brought up a related tool [the Levels of Emotional Awareness Scale–LEAS; cf. (126)] to evaluate the individuals' acquisition of emotional awareness from a developmental and ontogenetic perspective. The LEAS is often used as a “reverse control tool” measure for alexithymia and it is inversely correlated with TAS (115, 127, 128). Moreover, a specific theoretical contribution by Lane, Schwartz et al. to alexithymia research derives from their conceptualization of alexithymia as an emotional equivalent of blindsight (129) and as a form of “affective agnosia” (130, 131).

Further analysis of the documents within this cluster shows that between the ‘90s and the early 2000s many empirical studies were not interested in the condition of alexithymia *per se* but they used the construct of alexithymia in conjunction with others to investigate how emotions are processed. This hypothesis is supported by the presence of a set of highly cited articles referring to instruments which measure people's emotion processing capacities, mainly the Interpersonal Reactivity Index [IRI, (132)], the Positive and Negative Affect Schedule [PANAS, (133)], and the Difficulties in Emotion Regulation Scale (66). A number of studies focus in particular on emotion regulation, as witnessed by the position held by Gratz and Roemer (66), first document for burst strength within the cluster, and by the presence of other publications exploring the relationship between emotion regulation strategies and alexithymia (121) or well-being (134). Lastly, we might point out that within this cluster lies the paper referring to the Bermond-Vorst Alexithymia Questionnaire [BVAQ, (15)]. This self-report instrument was developed to operationalize the modularist conception of alexithymia put forward by its authors. Indeed, in Bermond and Vorst's view, we should distinguish between two different types of alexithymia. These derive from the disruption of different neural structures which support, respectively, the cognitive and the affective component of our emotional capacities. The BVAQ is currently used in conjunction with TAS in many empirical researches, although the latter is still the most used and reliable tool.

In summary, the research included in cluster #3 reflect the cognitive turn in alexithymia research which started from the mid-1990s. The articles belonging to this cluster employ a variety of cognitive tasks and neuroimaging techniques to explore how people with high alexithymia process emotion information in multiple domains (linguistic, visual etc.). Also the studies that introduce new assessment tools for alexithymia (i.e., the BVAQ) or examine alexithymia in conjunction with parallel constructs (i.e., emotional intelligence, emotion regulation and theory of mind) adopt a cognitive and neuroscientific approach.

#### Cluster #4: “Autism Spectrum Disorder”

This cluster represents the latest trends in alexithymia research and describes an ongoing line of research. The title “Autism Spectrum Disorder” aptly reflects the main point investigated by this cluster of papers, i.e., the comorbidity between alexithymia and Autism Spectrum Disorder (24). This explains also why the DSM-V and DSM-IV have high citation bursts within the cluster; in fact, they are cited mainly to define the condition of ASD. The same rationale applies for the three publications by Baron-Cohen et al. presenting the revised version of the “Reading the Mind in the Eyes” test (135) and the self-report Autism-Spectrum Quotient scale [AQ, (136)] and the self-report Empathy Quotient [EQ, (137)]. Among the main hypotheses investigated in the most influential articles, at least two are worth mentioning. The first one assumes that emotional impairments in ASD are due to the co-occurrence of alexithymia, rather than being a peculiar feature of ASD itself (24, 31, 75). The second one puts forward the idea that alexithymia and, by extension, poor emotional awareness are associated with deficits in visceroception or interoceptive ability (71, 138). Some influential publications in this cluster provide a definition of the neural structures supporting the interoceptive ability (139, 140) measured mainly through the heartbeat detection task. Other influential studies focus on methodological problems related to the measurement of the interoceptive ability (141) and provide theoretical models and methodological solutions to its study in conjunction with alexithymia (142–144). Finally, few recent publications combine the two hypotheses previously mentioned, suggesting that alexithymia could be conceived as a general deficit in interoception (145) and that the alexithymic traits or, more generally, the affective symptoms of people with ASD could be explained by the interoceptive difficulties they experience (146, 147).

In summary, Cluster #4 collects the most recent trends of research which are centered on the relationship between alexithymia and ASD. More specifically, the studies included in this group suggest that alexithymia plays a role as for the socio-communicative deficits exhibited by people suffering from ASD.

### Major Disciplinary Domains Found via JCA

Typically, journals are the point of reference of specific research communities: they do not only focus on particular subjects, but they also share a common perspective on them as well as preferred methods for their investigation. To scrutinize which journals published salient research on alexithymia in which periods is relevant to understand how this construct was approached by different research communities in different times. Therefore, JCA was applied to identify journals cited together in one paper.

Journal titles reported in Table 3 revealed something relevant concerning the conceptual history of alexithymia: they showed that the concept originated in the field of psychiatry and that it was relevant especially for psychosomatic research (see the title of journals with the earliest burst begin and the longest burst duration). These fields remained crucial for the research on alexithymia for at least 25 years. Then, these publication venues stopped their citation burst around 2002 and 2008. Indeed, almost none of the journals in the list of the top 30 for burst strength (Table 3) had an active burst process between 2008 and 2014, suggesting that this was a period of transition, characterized by a plurality of editorial venues addressing the research on alexithymia. Subsequently, from 2014, we found new journals whose citation burstness was still active in 2020 and it is therefore expected to continue with a consequent increase in burst strength index. Titles of the journals with the most recent citation bursts showed quite clearly that alexithymia has become a subject of interest as a different research area. Aspects related with psychosomatics and psychotherapy—which were dominant in the older studies published on this subject—were not mentioned anymore after 2014, indicating a shift in the research focus of the field. On the other hand, neuroscience was the main disciplinary domain of recent journals with a high burst strength (see *Social Cognitive and Affective Neuroscience, Frontiers in Human Neuroscience, Neuroscience & Biobehavioral Reviews*). This suggests that the neurobiological dimension of alexithymia has piqued the interest of researchers and that the field is actively working toward achieving a multifarious understanding of the condition. Moreover, the burst of journals covering a multitude of topics in the psychological sciences (*Frontiers in Psychology*) or, even, the presence of multidisciplinary journals such as *PLOS ONE* and *Scientific Reports* suggests that alexithymia is no longer a narrow niche subject of study. In fact, in this period the construct of alexithymia has become relevant for personality assessment as well as for research on emotional regulation/dysregulation. This is the reason why alexithymia is the object of growing attention by a wider, interdisciplinary scientific community represented by researchers and clinicians active in fields like psychology, psychiatry, medicine, and neuroscience.

## Conclusions

Any scientific domain–as well as any knowledge domain in general–subtends objective relations between different actors: i.e., institutions, authors, and journals. Network analysis represents an opportunity to computationally analyze and graphically represent the relations that characterize specific domains of knowledge.

Here we addressed the construct of alexithymia using co-citation analysis. Although the visibility of a paper is not a sufficient condition to assess its quality and relevance, it would at least be a necessary condition for gaining authority and for exercising an influence in a field. We applied this method to explore the macroscopic changes that occurred in this first half century of research on alexithymia. Specifically, our approach highlights the major thematic and methodological shifts that occurred within the community interested in alexithymia construct. Document Co-citation Analysis (DCA) and Journal Co-citation Analysis (JCA) suggest that the construct of alexithymia experienced a gradual conceptual and disciplinary shift. The analysis of the clusters shows that the body of research on alexithymia might be divided into three temporarily and logically distinct coarse units.

1. The first unit includes all the early articles published in the so-called “Pre-TAS era,” during which the researchers worked at a definition of the construct. The studies of this phase belong mainly to the area of psychosomatic medicine, psychiatry, and psychoanalytic treatment.
2. The second unit is centered around the activity of the “Toronto Group.” This includes the papers aimed at developing a standardized measurement of the construct and at establishing the differences and the overlaps of the TAS-20 with pre-existing constructs such as those of personality, depression etc.
3. The third unit comprises the studies of the “Post-TAS” era; their goal is to investigate the construct of alexithymia from the point of view of cognitive science and cognitive neuroscience as well as with behavioral and neurophysiological methods.

The multifactorial definition of alexithymia construct proposed by the Toronto Group has established a watershed within the history of alexithymia. The development of a standardized and easy to use tool such as the self-reported TAS-20 has been a driving force for empirical research, especially in the cognitive and neuroscientific domain. This shift in disciplines co-occurred with a modification in the conceptualization of the construct. Specifically, the interpersonal dimension of alexithymia, from peripheral and collateral aspects of the construct, has become the main focus of research. Indeed, the corpus of early publications is represented by cluster #1, which is focused on the recognition of one's own subjective feelings and therefore on the success of insight-oriented psychoanalytical treatment. In contrast, the research post TAS-20, represented by publications inside cluster #3 (renamed by us “Emotion Information Processing) and #4 (“Autism Spectrum Disorder”), focuses on the recognition of external emotional stimuli. This is evident based on the many studies in clusters #3 studying the deficits of individuals with high alexithymia to recognize emotional facial expressions or emotional images. This shift becomes even more evident in cluster #4, where the condition of alexithymia is primarily associated with a deficit in empathy and theory of mind in people with Autism Spectrum Disorder.

This change in intellectual thought concerning the conceptualization of alexithymia has been fostered by the new psychometric tools and by the experimental designs applied in the field of cognitive science. First, the TAS-20 implicitly modified the conceptualization of the construct by considering only three of the original four facets of the construct. Indeed, the TAS-20 measures (i) the difficulty identifying feelings, (ii) the difficulty describing feelings, and (iii) the externally-oriented thinking, while (iv) impoverished fantasy life has been excluded for psychometric reasons. Secondly, the development of the Toronto Alexithymia Scale gave new impetus to the research on tools to assess alexithymia. Independently from their supportive or skeptical attitude toward the TAS-20, a number of researchers explored the potentialities and the limits of this scale, comparing it with instruments measuring related constructs such as depression, anxiety, personality or emotional intelligence. The availability of a highly standardized and easy to administer instrument to assess alexithymia allowed researchers to explore this condition in various experimental settings by the use of behavioral and neurophysiological techniques. Consequently, this line of research is not any more restricted to the field of psychodynamics and psychosomatics, but has been expanding to a much larger community. Indeed, cognitive science and neuroscience are becoming increasingly pivotal in the field. The more influential publications in cluster #3 and #4 testify to this trend: most of them employ neuroimaging techniques and other neurophysiological measures and address issues concerning the neural basis of emotional processing. The same trend is confirmed by Journal Co-citation Analysis, and in particular by the journals with the strongest recent citation bursts (e.g., *Social Cognitive & Affective Neuroscience, Frontiers in Human Neuroscience, Neuroscience & Biobehavioral Reviews*). The evolution in time of the publications on alexithymia confirms a prediction made by Taylor and Bagby (70) at the end of their review of the “New trends in Alexithymia” at that time: “During the past 10 years alexithymia research has advanced and broadened considerably to include a wide spectrum of methodologies and experimental techniques that offer a significant challenge to alexithymia researchers. The pace of research will likely gain momentum as a result of current trends and increasing collaboration with investigators in other disciplines. The new methods and techniques must be embraced for the field of research to further increase understanding of the alexithymia construct and its association with physical and mental illness” [(70), p. 75]. Indeed, as Taylor and Bagby called for, new methods and techniques were embraced by the alexithymia research and, as a result, this became an interdisciplinary field intersecting a number of disciplines and issues concerning not only clinical psychology but also cognitive sciences and neurosciences.

Yet, some limitations of this study due to methodological problems should be noted.

First, we excluded from our pool of documents all those written in languages other than English (460 studies over an initial pool of 5,390). The use of WoS generates a linguistic bias for a scientometric study since the majority of the journals indexed in WoS are in English; only a small percentage of journals in other languages are included in this database. By excluding the documents written in languages other than English, we probably amplified this bias. However, the language is not irrelevant for the impact of a study: especially in the last decades, with the exception of particular fields/topics, publications written in languages other than English have mostly only a national audience and become relevant for international research only when they are discussed by other publications in English. It is plausible that publications in languages other than English did not catch the attention of a large scientific community; in this case, they would still be invisible in the networks built using DCA and JCA which, by definition, capture only macroscopic trends in research.

Secondly, the use of WoS as reference database also gives rise to a subtler bias related with the fact that psychoanalytic research–like the research in the field of humanities–is presented primarily in books, book chapters and journals that for the most part are not included in WoS or in other bibliometric databases. This applies especially to the past, but in part it still holds true today. Relying on the results we have achieved through our analysis, it seems that psychoanalysis gave a central contribution to the development of alexithymia only until the early'80s, which corresponds to the early years of the history of the construct. According to our scientometric study, it appears that later in time the psychoanalytic tradition gives up its interest in this construct. This is not the case. For example, McDougall (148–150) put forward relevant hypotheses on the etiology of alexithymia, considering alexithymic people as “disaffected” individuals: in her viewpoint alexithymic traits result from the “manifestation of defensives structures of a psychotic kind.” McDougall's perspective put forward an influential hypothesis on the psychogenesis of alexithymia, and yet in our psychometric analysis her name and her work do not appear to be relevant for the literature on alexithymia. The same applies for Bucci who developed an influential view on alexithymia on the basis of her Multiple Code Theory. In the perspective she proposes, human experience—including, in particular, emotional experience—is processed at three different levels of symbolizations and alexithymia is a disorder related with this processing (151, 152). Even though these researches do not stand out in our analysis, explicitly or otherwise, they played de facto a relevant role not only with respect to the improvement of the comprehension of alexithymia but also to making explicit its relevance for the cognitive and neuroscientific research [for a discussion on the importance of these authors cf. e.g., (153, 154)].

## Data Availability Statement

The raw data supporting the conclusions of this article will be made available by the authors, without undue reservation.

## Author Contributions

GG contributed to conceiving the study, created the dataset, conducted the analyses, and contributed to literature review and writing the article. AB contributed to the analyses and revising the paper. SD and LP conceived the study, contributed to the literature review, results interpretation, and writing the article. VA contributed to the study design and revising the article. GE conceived the study, obtained funding, and revised the paper. All authors contributed to the article and approved the submitted version.

## Conflict of Interest

The authors declare that the research was conducted in the absence of any commercial or financial relationships that could be construed as a potential conflict of interest.
