# Supplementary material for: A Scientometric Review of Alexithymia: Mapping Thematic and Disciplinary Shifts in Half a Century of Research
Source: Front Psychiatry. 2020 Dec 10;11:611489. doi: 10.3389/fpsyt.2020.611489 (PMC7758403; doi:10.3389/fpsyt.2020.611489)
Supplement: Supplementary file 1 [file Data_Sheet_1.pdf]

Supplementary Material

Supplementary Figure 1. Number of publications (blue line) per year retrieved from the Web of Science Core Collection selecting the topic “Alexithymia OR Alexithymic”. The exponential trendline is presented in red colour. On the horizontal axis: years (from the most recent). Vertical axis: number of records retrieved.

Supplementary Table 1 Number of publications for Document Types and Top 20 Authors, Organizations, Countries/ Regions for number of publications. Values are computed from the pool of 4930 publications in English language retrieved from the Web of Science Core Collection selecting the topic "Alexithymia OR Alexithymic".

**Document Types**	**Number of Publications**
ARTICLE	3863
MEETING ABSTRACT	657
REVIEW	182
PROCEEDINGS PAPER	138
LETTER	83
EARLY ACCESS	58
EDITORIAL MATERIAL	46
BOOK REVIEW	19
BOOK CHAPTER	12
NOTE	8
CORRECTION	7
CORRECTION ADDITION	1
NEWS ITEM	1
REPRINT	1
**Authors**	**Number of Publications**
TAYLOR GJ	91
BAGBY RM	83
LUMINET O	70
PARKER JDA	62
FUKUNISHI I	56
JOUKAMAA M	55
PORCELLI P	49
GRABE HJ	45
BIRD G	42
VIINAMAKI H	42
HONKALAMPI K	41
LUMLEY MA	40

LANE RD	39
HINTIKKA J	38
LYVERS M	37
THORBERG FA	36
DIMAGGIO G	35
CORCOS M	33
LOAS G	32
WISE TN	29

**Organizations**	**Number of Publications**
UNIV TORONTO	148
KINGS COLL LONDON	99
UNIV ROMA LA SAPIENZA	95
UCL	87
MT SINAI HOSPITAL	80
CATHOLIC UNIV LOUVAIN	71
UNIV BOLOGNA	60
UNIV TURIN	59
UNIV PADUA	55
KUOPIO UNIV HOSP	53
WAYNE STATE UNIV	53
SAPIENZA UNIV ROME	52
LEIDEN UNIV	49
UNIV TURKU	49
TILBURG UNIV	48
UNIV TAMPERE	48
UNIV AMSTERDAM	45
UNIV CALIF LOS ANGELES	44
UNIV OSLO	43
YORK UNIVERSITY	43

**Countries/Regions**	**Number of Publications**
USA	1155
ITALY	787

ENGLAND	455
CANADA	385
GERMANY	385
NETHERLANDS	257
FRANCE	250
BELGIUM	199
AUSTRALIA	198
FINLAND	189
TURKEY	174
JAPAN	158
SWEDEN	115
PEOPLES R CHINA	114
SPAIN	103
SWITZERLAND	102
IRAN	78
NORWAY	72
ISRAEL	71
POLAND	61

Supplementary Table 2 References with a citation burst of at least two years computed via document co-citation analysis (DCA). References are ordered by burst strength.

Burst Begin	Burst End	Strength	Year	References (DCA)
1988	2003	70.1089	1985	TAYLOR GJ, 1985, PSYCHOTHER PSYCHOSOM, V44, P191, DOI 10.1159/000287912
1985	2003	64.3921	1984	TAYLOR GJ, 1984, AM J PSYCHIAT, V141, P725
2015	2020	58.3792	2013	**AmericanPsychiatricAssociation, 2013, DIAGN STAT MAN MENT, V0, P0
1981	2001	57.7409	1980	APFEL RJ, 1979, PSYCHOTHER PSYCHOSOM, V32, P180, DOI 10.1159/000287386
1989	1998	54.2461	1988	TAYLOR GJ, 1988, PSYCHOSOM MED, V50, P500, DOI 10.1097/00006842-198809000-00006
1988	2002	49.1502	1986	BAGBY RM, 1986, PSYCHOTHER PSYCHOSOM, V45, P207
1998	2012	39.5262	1994	**AmericanPsychiatricAssociation, 1994, DIAGN STAT MAN MENT, V0, P0
1988	2001	38.0664	1988	BAGBY RM, 1988, J PSYCHOSOM RES, V32, P107, DOI 10.1016/0022-3999(88)90094-3
2016	2020	37.4279	2004	Gratz KL, 2004, J PSYCHOPATHOL BEHAV, V26, P41, DOI 10.1023/B:JOBA.0000007455.08539.94
1981	2001	37.3512	1980	KRYSTAL H, 1979, AM J PSYCHOTHER, V33, P17
1981	1998	36.8877	1980	KLEIGER JH, 1980, PSYCHOTHER PSYCHOSOM, V34, P17, DOI 10.1159/000287442
1982	2003	36.2049	1980	FREYBERGER H, 1977, PSYCHOTHER PSYCHOSOM, V28, P337, DOI 10.1159/000287080
1983	1998	35.7896	1981	LESSER IM, 1981, PSYCHOSOM MED, V43, P531, DOI 10.1097/00006842-198112000-00009
1981	2001	33.6823	1980	SIFNEOS PE, 1972, SHORT TERM PSYCHOTHE, V0, P0

1992	2000	33.5943	1990	TAYLOR GJ, 1990, CAN J PSYCHIAT, V35, P290, DOI 10.1177/070674379003500402
2006	2012	32.3127	2004	Taylor GJ, 2004, PSYCHOTHER PSYCHOSOM, V73, P68, DOI 10.1159/000075537
2015	2020	31.903	2011	Herbert BM, 2011, J PERS, V79, P1149, DOI 10.1111/j.1467-6494.2011.00717.x
2015	2020	31.903	2013	Bird G, 2013, TRANSL PSYCHIAT, V3, P0, DOI 10.1038/tp.2013.61
1985	2006	31.5378	1980	NEMIAH JC, 1970, MODERN TRENDS PSYCHO, V2, P26
1995	2006	30.3956	1993	PARKER JDA, 1993, EUR J PERSONALITY, V7, P221, DOI 10.1002/per.2410070403
1998	2010	29.9756	1997	Taylor G J, 1997, DISORDERS AFFECT REG, V0, P0
1994	2002	29.4723	1992	TAYLOR GJ, 1992, J PSYCHOSOM RES, V36, P417, DOI 10.1016/0022-3999(92)90002-J
2013	2020	28.6679	2010	Bird G, 2010, BRAIN, V133, P1515, DOI 10.1093/brain/awq060
1989	2001	28.2653	1988	KRYSTAL H, 1988, INTEGRATION SELF HEA, V0, P0
1982	1992	27.8224	1981	BLANCHARD EB, 1981, PSYCHOTHER PSYCHOSOM, V35, P64, DOI 10.1159/000287479
2014	2020	27.7846	2012	Grynberg D, 2012, PLOS ONE, V7, P0, DOI 10.1371/journal.pone.0042429
1992	2001	27.54	1991	TAYLOR GJ, 1991, PSYCHOSOMATICS, V32, P153, DOI 10.1016/S0033-3182(91)72086-0
1992	1999	27.445	1990	TAYLOR GJ, 1990, AM J PSYCHIAT, V147, P1228
2015	2020	27.3173	2013	Cook R, 2013, PSYCHOL SCI, V24, P723, DOI 10.1177/0956797612463582
1989	2002	26.8049	1987	**AmericanPsychiatricAssociation, 1987, DIAGN STAT MAN MENT, V0, P0

1993	2007	26.7443	1992	BOURKE MP, 1992, BRIT J PSYCHIAT, V161, P240, DOI 10.1192/bjp.161.2.240
1989	2000	26.6103	1988	BAGBY RM, 1988, PSYCHOTHER PSYCHOSOM, V50, P29
1988	2001	26.5637	1986	KRYSTAL JH, 1986, PSYCHOSOM MED, V48, P84, DOI 10.1097/00006842-198601000-00007
2016	2020	26.0635	2015	Li SW, 2015, PSYCHIAT RES, V227, P1, DOI 10.1016/j.psychres.2015.02.006
1995	2005	26.0002	1993	SCHMIDT U, 1993, COMPR PSYCHIAT, V34, P54, DOI 10.1016/0010-440X(93)90036-4
1993	2007	25.7742	1991	PARKER JDA, 1991, COMPR PSYCHIAT, V32, P387, DOI 10.1016/0010-440X(91)90015-5
1995	2002	25.0193	1994	TAYLOR GJ, 1994, NEW TRENDS EXPT CLIN, V10, P61
1991	2001	24.4337	1988	HAVILAND MG, 1988, PSYCHOTHER PSYCHOSOM, V50, P81, DOI 10.1159/000288104
1984	1999	23.6744	1982	Krystal H, 1982, Int J Psychoanal Psychother, V9, P353
1990	2001	23.6113	1989	PARKER JDA, 1989, COMPR PSYCHIAT, V30, P434, DOI 10.1016/0010-440X(89)90009-6
2015	2020	23.3291	2011	Ogrodniczuk JS, 2011, PSYCHIAT RES, V190, P43, DOI 10.1016/j.psychres.2010.04.026
1992	2004	23.2824	1991	HENDRYX MS, 1991, J PERS ASSESS, V56, P227, DOI 10.1207/s15327752jpa5602_4
1991	2000	23.2261	1989	ZEITLIN SB, 1989, AM J PSYCHIAT, V146, P1434
2000	2010	23.0943	1998	Lane RD, 1998, COMPR PSYCHIAT, V39, P377, DOI 10.1016/S0010-440X(98)90051-7
1985	1993	23.0822	1983	SMITH GR, 1983, AM J PSYCHIAT, V140, P99
1980	1991	21.9942	1980	SIFNEOS PE, 1977, PSYCHOTHER PSYCHOSOM, V28, P47, DOI 10.1159/000287043

1993	2003	21.7734	1990	WISE TN, 1990, COMPR PSYCHIAT, V31, P284, DOI 10.1016/0010-440X(90)90035-Q
2014	2020	21.6935	2013	van der Velde J, 2013, NEUROSCI BIOBEHAV R, V37, P1774, DOI 10.1016/j.neubiorev.2013.07.008
2016	2020	21.5636	2013	Hayes A F, 2013, INTRO MEDIATION MODE, V0, P0
2018	2020	21.5538	2016	Brewer R, 2016, ROY SOC OPEN SCI, V3, P0, DOI 10.1098/rsos.150664
2015	2020	21.4587	1999	Taylor G J, 1999, DISORDERS AFFECT REG, V0, P0
2003	2011	21.3573	2001	Joukamaa M, 2001, NORD J PSYCHIAT, V55, P123, DOI 10.1080/08039480151108561
2006	2013	21.3159	2004	Grabe HJ, 2004, AM J PSYCHIAT, V161, P1299, DOI 10.1176/appi.ajp.161.7.1299
1992	1998	21.1982	1990	BAGBY RM, 1990, J PSYCHOSOM RES, V34, P47, DOI 10.1016/0022-3999(90)90007-Q
1985	1996	21.0564	1980	NEMIAH JC, 1977, PSYCHOTHER PSYCHOSOM, V28, P199, DOI 10.1159/000287064
2013	2020	20.9273	2001	Baron-Cohen S, 2001, J CHILD PSYCHOL PSYC, V42, P241, DOI 10.1017/S0021963001006643
2004	2010	20.8764	1999	Luminet O, 1999, J PERS ASSESS, V73, P345, DOI 10.1207/S15327752JPA7303_4
1999	2010	20.5748	1996	Lumley MA, 1996, J PSYCHOSOM RES, V41, P505, DOI 10.1016/S0022-3999(96)00222-X
1982	1996	20.4902	1980	SIFNEOS PE, 1975, PSYCHOTHER PSYCHOSOM, V26, P65, DOI 10.1159/000286912
1995	2001	20.4678	1994	JIMERSON DC, 1994, PSYCHOSOM MED, V56, P90, DOI 10.1097/00006842-199403000-00002
2000	2011	20.2803	1996	Sifneos PE, 1996, AM J PSYCHIAT, V153, P137

1981	1989	20.1347	1980	KLEIGER JH, 1980, J NERV MENT DIS, V168, P465, DOI 10.1097/00005053-198008000-00003
1991	2001	20.0815	1988	HAVILAND MG, 1988, PSYCHOTHER PSYCHOSOM, V50, P164, DOI 10.1159/000288115
2012	2017	19.7466	2008	Mattila AK, 2008, PSYCHOSOM MED, V70, P716, DOI 10.1097/PSY.0b013e31816ffc39
2018	2020	19.5814	2016	Shah P, 2016, CORTEX, V81, P215, DOI 10.1016/j.cortex.2016.03.021
1996	2004	19.3116	1993	ZEITLIN SB, 1993, AM J PSYCHIAT, V150, P658
2001	2010	19.0851	2000	Honkalampi K, 2000, PSYCHOTHER PSYCHOSOM, V69, P303, DOI 10.1159/000012412
2012	2020	19.0221	2009	Thorberg FA, 2009, ADDICT BEHAV, V34, P237, DOI 10.1016/j.addbeh.2008.10.016
2012	2020	18.9389	2009	Swart M, 2009, PLOS ONE, V4, P0, DOI 10.1371/journal.pone.0005751
2000	2006	18.7681	1988	BECK AT, 1988, CLIN PSYCHOL REV, V8, P77, DOI 10.1016/0272-7358(88)90050-5
1985	1995	18.6122	1985	LESSER IM, 1985, NEW ENGL J MED, V312, P690, DOI 10.1056/NEJM198503143121105
2008	2013	18.5672	1986	BARON RM, 1986, J PERS SOC PSYCHOL, V51, P1173, DOI 10.1037/0022-3514.51.6.1173
2013	2020	18.3969	1983	DAVIS MH, 1983, J PERS SOC PSYCHOL, V44, P113, DOI 10.1037/0022-3514.44.1.113
2005	2011	18.3202	2003	Taylor GJ, 2003, J PSYCHOSOM RES, V55, P277, DOI 10.1016/S0022-3999(02)00601-3
2012	2020	18.2578	2003	Gross JJ, 2003, J PERS SOC PSYCHOL, V85, P348, DOI 10.1037/0022-3514.85.2.348
2014	2018	18.2566	2010	Reker M, 2010, CORTEX, V46, P658, DOI 10.1016/j.cortex.2009.05.008

1996	2008	18.196	1994	BERENBAUM H, 1994, PSYCHOSOM MED, V56, P353, DOI 10.1097/00006842-199407000-00011
1989	1996	18.175	1988	TAYLOR GJ, 1988, PSYCHIAT CLIN N AM, V11, P351
2007	2011	18.1357	2006	Saarijarvi S, 2006, PSYCHOTHER PSYCHOSOM, V75, P107, DOI 10.1159/000090895
2016	2020	18.1158	2010	Grynberg D, 2010, PERS INDIV DIFFER, V49, P845, DOI 10.1016/j.paid.2010.07.013
1981	1996	18.0841	1980	MARTY P, 1963, REV FRANCAISE PSYC S, V27, P1345
1980	1986	17.9623	1980	PIERLOOT R, 1977, PSYCHOTHER PSYCHOSOM, V28, P156, DOI 10.1159/000287058
1991	1996	17.6244	1988	ACKLIN MW, 1988, J NERV MENT DIS, V176, P343, DOI 10.1097/00005053-198806000-00003
2004	2008	17.4785	2001	Honkalampi K, 2001, PSYCHOTHER PSYCHOSOM, V70, P247, DOI 10.1159/000056262
2005	2015	17.4438	2003	Kano M, 2003, BRAIN, V126, P1474, DOI 10.1093/brain/awg131
2003	2010	17.4219	2000	Taylor GJ, 2000, CAN J PSYCHIAT, V45, P134, DOI 10.1177/070674370004500203
2003	2016	17.4194	2002	Berthoz S, 2002, AM J PSYCHIAT, V159, P961, DOI 10.1176/appi.ajp.159.6.961
2014	2017	17.1719	2013	Moriguchi Y, 2013, BIOPSYCHOSOC MED, V7, P0, DOI 10.1186/1751-0759-7-8
1988	2003	17.0098	1986	MARTIN JB, 1986, PSYCHOTHER PSYCHOSOM, V45, P66, DOI 10.1159/000287930
2010	2018	16.9968	2007	Lumley MA, 2007, J PERS ASSESS, V89, P230, DOI 10.1080/00223890701629698
2013	2020	16.8798	2009	Craig AD, 2009, NAT REV NEUROSCI, V10, P59, DOI 10.1038/nrn2555

2006	2012	16.807	2005	Bydlowski S, 2005, INT J EAT DISORDER, V37, P321, DOI 10.1002/eat.20132
1981	1990	16.7185	1980	LOLAS F, 1980, PSYCHOTHER PSYCHOSOM, V33, P139, DOI 10.1159/000287424
2016	2020	16.7067	1981	SCHANDRY R, 1981, PSYCHOPHYSIOLOGY, V18, P483, DOI 10.1111/j.1469-8986.1981.tb02486.x
1994	2003	16.6956	1993	KIRMAYER LJ, 1993, PSYCHOSOMATICS, V34, P41, DOI 10.1016/S0033-3182(93)71926-X
1992	2001	16.6422	1991	HAVILAND MG, 1991, J NERV MENT DIS, V179, P284, DOI 10.1097/00005053-199105000-00007
2012	2020	16.6407	2008	Silani G, 2008, SOC NEUROSCI-UK, V3, P97, DOI 10.1080/17470910701577020
2015	2020	16.4168	2002	Craig AD, 2002, NAT REV NEUROSCI, V3, P655, DOI 10.1038/nrn894
2015	2020	16.3343	2009	Levant RF, 2009, PSYCHOL MEN MASCULIN, V10, P190, DOI 10.1037/a0015652
2013	2017	16.2701	2007	Moriguchi Y, 2007, CEREB CORTEX, V17, P2223, DOI 10.1093/cercor/bhl130
2016	2020	16.2635	2015	Lane RD, 2015, NEUROSCI BIOBEHAV R, V55, P594, DOI 10.1016/j.neubiorev.2015.06.007
2015	2017	15.9999	2011	Tolmunen T, 2011, COMPR PSYCHIAT, V52, P536, DOI 10.1016/j.comppsych.2010.09.007
1984	1994	15.9947	1982	SHIPKO S, 1982, PSYCHOTHER PSYCHOSOM, V37, P193, DOI 10.1159/000287573
2005	2012	15.763	1998	Lane RD, 1998, J COGNITIVE NEUROSCI, V10, P525, DOI 10.1162/089892998562924
1995	2005	15.7255	1993	COCHRANE CE, 1993, INT J EAT DISORDER, V14, P219, DOI 10.1002/1098-108X(199309)14:2<219::AID-EAT2260140212>3.0.CO;2-G

1993	2003	15.5872	1992	TAYLOR GJ, 1992, PSYCHOTHER PSYCHOSOM, V57, P34, DOI 10.1159/000288571
2010	2016	15.5288	2008	Karlsson H, 2008, BRIT J PSYCHIAT, V192, P32, DOI 10.1192/bjp.bp.106.034728
2014	2018	15.4535	2008	Preacher KJ, 2008, BEHAV RES METHODS, V40, P879, DOI 10.3758/BRM.40.3.879
2018	2020	15.386	2015	Garfinkel SN, 2015, BIOL PSYCHOL, V104, P65, DOI 10.1016/j.biopsycho.2014.11.004
2008	2013	15.2367	2006	Bagby RM, 2006, PSYCHOTHER PSYCHOSOM, V75, P25, DOI 10.1159/000089224
1980	1996	15.2344	1980	NEMIAH JC, 1970, MODERN TRENDS PSYCHO, V2, P0
2003	2012	15.1492	2001	Luminet O, 2001, PSYCHOTHER PSYCHOSOM, V70, P254, DOI 10.1159/000056263
2009	2016	15.127	2006	Moriguchi Y, 2006, NEUROIMAGE, V32, P1472, DOI 10.1016/j.neuroimage.2006.04.186
2008	2015	15.1197	2001	Honkalampi K, 2001, PSYCHOSOMATICS, V42, P229, DOI 10.1176/appi.psy.42.3.229
2005	2014	15.0306	2001	Parker JDA, 2001, PERS INDIV DIFFER, V30, P107, DOI 10.1016/S0191-8869(00)00014-3
2013	2017	14.9357	1999	Berthoz S, 1999, EUR PSYCHIAT, V14, P372, DOI 10.1016/S0924-9338(99)00233-3
1981	1991	14.8883	1980	RUESCH J, 1948, PSYCHOSOM MED, V10, P134, DOI 10.1097/00006842-194805000-00002
2018	2020	14.8723	2017	Westwood H, 2017, J PSYCHOSOM RES, V99, P66, DOI 10.1016/j.jpsychores.2017.06.007
2017	2020	14.7753	2014	Cameron K, 2014, HARVARD REV PSYCHIAT, V22, P162, DOI 10.1097/HRP.0000000000000036
2013	2018	14.761	1996	Beck A, 1996, MANUAL BECK DEPRESSI, V0, P0

1980	1990	14.7246	1980	NEMIAH JC, 1975, PSYCHOTHER PSYCHOSOM, V26, P140, DOI 10.1159/000286923
2007	2011	14.7036	2005	Picardi A, 2005, PSYCHOTHER PSYCHOSOM, V74, P371, DOI 10.1159/000087785
1997	2003	14.6021	1994	SALMINEN JK, 1994, J PSYCHOSOM RES, V38, P681, DOI 10.1016/0022-3999(94)90020-5
2013	2020	14.3533	2005	Berthoz S, 2005, EUR PSYCHIAT, V20, P291, DOI 10.1016/j.eurpsy.2004.06.013
2018	2020	14.334	2013	**AmericanPsychiatricAssociation, 2013, DIAGNOSTIC STAT MANU, V0, P0
2014	2018	14.2923	2013	Kano M, 2013, BIOPSYCHOSOC MED, V7, P0, DOI 10.1186/1751-0759-7-1
2016	2020	14.2524	2013	Taylor GJ, 2013, J AM PSYCHOANAL ASS, V61, P99, DOI 10.1177/0003065112474066
2015	2018	14.2518	2004	Critchley HD, 2004, NAT NEUROSCI, V7, P189, DOI 10.1038/nn1176
2013	2020	14.1008	2012	Leweke F, 2012, PSYCHOPATHOLOGY, V45, P22, DOI 10.1159/000325170
2013	2017	13.9515	2011	Nicolo G, 2011, PSYCHIAT RES, V190, P37, DOI 10.1016/j.psychres.2010.07.046
1995	2004	13.8465	1993	PARKER JDA, 1993, PSYCHOTHER PSYCHOSOM, V59, P197, DOI 10.1159/000288664
2000	2007	13.7629	1999	Honkalampi K, 1999, PSYCHOTHER PSYCHOSOM, V68, P270, DOI 10.1159/000012343
2001	2011	13.7413	1996	Loas G, 1996, PSYCHOPATHOLOGY, V29, P139, DOI 10.1159/000284983
1986	1996	13.462	1982	MENDELSON G, 1982, PSYCHOTHER PSYCHOSOM, V37, P154, DOI 10.1159/000287568
2012	2017	13.3702	1997	First M B, 1997, STRUCTURED CLIN INTE, V0, P0

2011	2016	13.2014	2009	Borsci G, 2009, J AFFECT DISORDERS, V114, P208, DOI 10.1016/j.jad.2008.07.013
2001	2007	13.1313	1995	BACH M, 1995, PSYCHOTHER PSYCHOSOM, V64, P43, DOI 10.1159/000288989
1995	1999	13.0943	1992	KAUHANEN J, 1992, BEHAV MED, V18, P121, DOI 10.1080/08964289.1992.9936962
1981	1992	13.0249	1980	LESSER IM, 1979, GEN HOSP PSYCHIAT, V1, P256, DOI 10.1016/0163-8343(79)90027-6
2012	2017	12.901	2002	Guttman H, 2002, COMPR PSYCHIAT, V43, P448, DOI 10.1053/comp.2002.35905
2009	2011	12.8752	2008	Grabe HJ, 2008, PSYCHOTHER PSYCHOSOM, V77, P189, DOI 10.1159/000119739
2018	2020	12.827	1998	Gross J J, 1998, REV GEN PSYCHOL, V2, P271, DOI DOI 10.1037/1089-2680.2.3.271
2009	2015	12.7603	2003	Coffey E, 2003, COGNITION EMOTION, V17, P671, DOI 10.1080/02699930302304
1999	2006	12.7588	1996	Haviland MG, 1996, J PERS ASSESS, V66, P116, DOI 10.1207/s15327752jpa6601_9
2003	2007	12.7325	1995	DEGROOT JM, 1995, COMPR PSYCHIAT, V36, P53, DOI 10.1016/0010-440X(95)90099-H
2010	2020	12.7081	1983	Spielberger C D, 1983, MANUAL STATE TRAIT A, V0, P0
1997	2002	12.6633	1990	HYER L, 1990, J CLIN PSYCHIAT, V51, P243
2009	2016	12.6514	2000	**AmericanPsychiatricAssociation, 2000, DIAGN STAT MAN MENT, V0, P0
1999	2010	12.6417	1996	Kauhanen J, 1996, J PSYCHOSOM RES, V41, P541, DOI 10.1016/S0022-3999(96)00226-7
2004	2010	12.6118	2001	Lundh LG, 2001, J PERS, V69, P483, DOI 10.1111/1467-6494.00153

1980	1991	12.5582	1980	McDougall J, 1974, INT REV PSYCHOANAL, V1, P437
2005	2013	12.531	2000	Haviland MG, 2000, PSYCHOSOMATICS, V41, P385, DOI 10.1176/appi.psy.41.5.385
1985	1999	12.4503	1981	TAYLOR G, 1981, CAN J PSYCHIAT, V26, P470, DOI 10.1177/070674378102600706
2003	2007	12.4108	1999	Porcelli P, 1999, PSYCHOTHER PSYCHOSOM, V68, P263, DOI 10.1159/000012342
1988	1995	12.36	1986	BAGBY RM, 1986, COMPR PSYCHIAT, V27, P287, DOI 10.1016/0010-440X(86)90004-0
2015	2020	12.3519	2009	Gulec H, 2009, KLIN PSIKOFARMAKOL B, V19, P214
1980	1995	12.3163	1980	FLANNERY JG, 1977, PSYCHOTHER PSYCHOSOM, V28, P133, DOI 10.1159/000287055
2015	2018	12.2711	2013	Nowakowski ME, 2013, J EAT DISORD, V1, P0, DOI 10.1186/2050-2974-1-21
2008	2015	12.2298	2005	Mantani T, 2005, BIOL PSYCHIAT, V57, P982, DOI 10.1016/j.biopsych.2005.01.047
2003	2010	12.098	2001	Loas G, 2001, J PSYCHOSOM RES, V50, P255, DOI 10.1016/S0022-3999(01)00197-0
2008	2012	12.0978	1997	Loas G, 1997, COMPR PSYCHIAT, V38, P296, DOI 10.1016/S0010-440X(97)90063-8
2005	2011	12.0436	2003	Porcelli P, 2003, PSYCHOSOM MED, V65, P911, DOI 10.1097/01.PSY.0000089064.13681.3B
2007	2015	11.9754	2003	Parker JDA, 2003, J PSYCHOSOM RES, V55, P269, DOI 10.1016/S0022-3999(02)00578-0
2014	2016	11.9457	2001	Hintikka J, 2001, COMPR PSYCHIAT, V42, P234, DOI 10.1053/comp.2001.23147
2000	2005	11.9116	1997	Deary IJ, 1997, PERS INDIV DIFFER, V22, P551, DOI 10.1016/S0191-8869(96)00229-2

2004	2015	11.8814	2001	Bankier B, 2001, PSYCHOSOMATICS, V42, P235, DOI 10.1176/appi.psy.42.3.235
2014	2017	11.8713	2008	Frewen PA, 2008, J TRAUMA STRESS, V21, P243, DOI 10.1002/jts.20320
2004	2012	11.8023	1995	SALOVEY P, 1995, EMOTION DISCLOSURE H, V0, P125, DOI DOI 10.1037/10182-006
2018	2020	11.7916	2016	Taylor GJ, 2016, NEUROSCI BIOBEHAV R, V68, P1006, DOI 10.1016/j.neubiorev.2016.05.025
1985	1990	11.765	1984	GARDOS G, 1984, COMPR PSYCHIAT, V25, P278, DOI 10.1016/0010-440X(84)90058-0
2002	2011	11.6853	1996	Berenbaum H, 1996, J PSYCHOSOM RES, V41, P585, DOI 10.1016/S0022-3999(96)00225-5
2018	2020	11.6765	2017	Murphy J, 2017, DEV COGN NEUROS-NETH, V23, P45, DOI 10.1016/j.dcn.2016.12.006
1994	1999	11.6242	1991	KAUHANEN J, 1991, PSYCHOTHER PSYCHOSOM, V56, P247, DOI 10.1159/000288563
1998	2000	11.6186	1996	Lumley MA, 1996, PSYCHOSOM MED, V58, P197, DOI 10.1097/00006842-199605000-00001
2007	2013	11.6123	2005	Subic-Wrana C, 2005, PSYCHOSOM MED, V67, P483, DOI 10.1097/01.psy.0000160461.19239.13
2005	2013	11.5958	2002	Kooiman CG, 2002, J PSYCHOSOM RES, V53, P1083, DOI 10.1016/S0022-3999(02)00348-3
1995	2000	11.5502	1994	COHEN K, 1994, J PSYCHOSOM RES, V38, P119, DOI 10.1016/0022-3999(94)90085-X
2005	2010	11.4787	2000	Lumley MA, 2000, J PSYCHOSOM RES, V49, P51, DOI 10.1016/S0022-3999(00)00161-6
2002	2004	11.4102	1999	Mayer JD, 1999, INTELLIGENCE, V27, P267, DOI 10.1016/S0160-2896(99)00016-1

1985	1992	11.358	1983	DEMERSDESROSIERS LA, 1983, PSYCHOTHER PSYCHOSOM, V39, P65, DOI 10.1159/000287724
1985	1992	11.358	1983	LESSER IM, 1983, AM J PSYCHIAT, V140, P1305
1988	1994	11.3309	1987	ACKLIN MW, 1987, J PERS ASSESS, V51, P462, DOI 10.1207/s15327752jpa5103_13
2016	2018	11.3248	2002	Fonagy P, 2002, AFFECT REGULATION ME, V0, P0
2001	2010	11.2266	2000	Beresnevaite M, 2000, PSYCHOTHER PSYCHOSOM, V69, P117, DOI 10.1159/000012378
1980	1988	11.089	1980	NEMIAH JC, 1976, MODERN TRENDS PSYCHO, V3, P0
2015	2016	11.0383	2004	Paivio SC, 2004, CHILD ABUSE NEGLECT, V28, P339, DOI 10.1016/j.chiabu.2003.11.018
2016	2020	10.9705	2009	Machin GC, 2009, PERS INDIV DIFFER, V46, P412, DOI 10.1016/j.paid.2008.11.010
2005	2009	10.9186	2000	Corcos M, 2000, PSYCHIAT RES, V93, P263, DOI 10.1016/S0165-1781(00)00109-8
2002	2007	10.9163	1989	Salovey Peter, 1989, IMAGINATION COGNITIO, V9, P185, DOI DOI 10.2190/DUGG-P24E-52WK-6CDG
2018	2020	10.8361	2001	Baron-Cohen S, 2001, J AUTISM DEV DISORD, V31, P5, DOI 10.1023/A:1005653411471
2013	2015	10.7738	2003	De Gucht V, 2003, J PSYCHOSOM RES, V54, P425, DOI 10.1016/S0022-3999(02)00467-1
1981	1986	10.6816	1980	KLEIGER JH, 1980, PSYCHOTHER PSYCHOSOM, V34, P25, DOI 10.1159/000287443
1986	1989	10.6793	1984	MARTIN JB, 1984, PSYCHOTHER PSYCHOSOM, V41, P145, DOI 10.1159/000287802
2018	2020	10.6139	2018	Murphy J, 2018, J EXP PSYCHOL GEN, V147, P398, DOI 10.1037/xge0000366

1997	2003	10.5096	1997	Fukunishi I, 1997, PSYCHOL REP, V80, P787, DOI 10.2466/pr0.1997.80.3.787
2011	2013	10.5068	2006	Luminet O, 2006, J RES PERS, V40, P713, DOI 10.1016/j.jrp.2005.09.001
2002	2011	10.4744	1997	Lane RD, 1997, BIOL PSYCHIAT, V42, P834, DOI 10.1016/S0006-3223(97)00050-4
2002	2015	10.405	2000	Lane RD, 2000, PSYCHOSOM MED, V62, P492, DOI 10.1097/00006842-200007000-00007
1983	1990	10.3545	1980	BORENS R, 1977, PSYCHOTHER PSYCHOSOM, V28, P193, DOI 10.1159/000287063
2018	2020	10.3193	2004	Hill E, 2004, J AUTISM DEV DISORD, V34, P229, DOI 10.1023/B:JADD.0000022613.41399.14
2011	2014	10.305	1996	Taylor GJ, 1996, J PSYCHOSOM RES, V41, P561, DOI 10.1016/S0022-3999(96)00224-3
2005	2008	10.2836	2000	Taylor GJ, 2000, HDB EMOTIONAL INTELL, V0, P40
1993	1996	10.2447	1991	RUBINO IA, 1991, BRIT J MED PSYCHOL, V64, P385, DOI 10.1111/j.2044-8341.1991.tb01675.x
2013	2015	10.2417	2002	Adolphs R, 2002, CURR OPIN NEUROBIOL, V12, P169, DOI 10.1016/S0959-4388(02)00301-X
1986	1992	10.2165	1983	BARSKY AJ, 1983, AM J PSYCHIAT, V140, P273
1998	2011	10.1752	1996	Lane RD, 1996, PSYCHOSOM MED, V58, P203, DOI 10.1097/00006842-199605000-00002
2016	2020	10.1568	2014	Bird G, 2014, NEUROSCI BIOBEHAV R, V47, P520, DOI 10.1016/j.neubiorev.2014.09.021
2015	2020	10.1149	1999	Hu LT, 1999, STRUCT EQU MODELING, V6, P1, DOI 10.1080/10705519909540118
1999	2006	10.098	1994	COX BJ, 1994, J PSYCHOSOM RES, V38, P523, DOI 10.1016/0022-3999(94)90049-3

2011	2016	10.091	2007	Kano M, 2007, PAIN, V132, P252, DOI 10.1016/j.pain.2007.01.032
2018	2020	10.0827	2016	Di Tella M, 2016, CURR RHEUMATOL REP, V18, P0, DOI 10.1007/s11926-016-0592-x
2018	2020	10.0827	2001	Barrett LF, 2001, COGNITION EMOTION, V15, P713, DOI 10.1080/02699930143000239
2018	2020	10.0827	1995	Patton JH, 1995, J CLIN PSYCHOL, V51, P768, DOI 10.1002/1097-4679(199511)51:6<768::AID-JCLP2270510607>3.0.CO;2-1
2018	2020	10.0827	1998	Gross JJ, 1998, J PERS SOC PSYCHOL, V74, P224, DOI 10.1037/0022-3514.74.1.224
2013	2014	10.0725	2006	Waller E, 2006, INT REV PSYCHIATR, V18, P13, DOI 10.1080/09540260500466774
2003	2008	10.0088	1992	Costa P T, 1992, REVISED NEO PERSONAL, V0, P0
1982	1997	9.9843	1980	HOPPE KD, 1977, PSYCHOTHER PSYCHOSOM, V28, P148, DOI 10.1159/000287057
2017	2018	9.9163	2014	Goerlich-Dobre KS, 2014, NEUROPSYCHOLOGIA, V53, P284, DOI 10.1016/j.neuropsychologia.2013.12.006
2015	2016	9.8022	2010	Karukivi M, 2010, J AFFECT DISORDERS, V125, P383, DOI 10.1016/j.jad.2010.02.126
1991	1994	9.7746	1986	SIFNEOS PE, 1986, PSYCHOTHER PSYCHOSOM, V45, P161, DOI 10.1159/000287942
2008	2012	9.7625	2005	Cleland C, 2005, J PSYCHOSOM RES, V58, P299, DOI 10.1016/j.jpsychores.2004.11.002
2008	2013	9.7414	2003	Larsen JK, 2003, J PSYCHOSOM RES, V54, P533, DOI 10.1016/S0022-3999(02)00466-X
2018	2020	9.7388	2009	Leising D, 2009, J RES PERS, V43, P707, DOI 10.1016/j.jrp.2009.03.009
2005	2011	9.7165	2000	TAYLOR GJ, 2000, HDB EMOTIONAL INTELL, V0, P301

2005	2008	9.7118	1997	BAGBY RM, 1997, DISORDERS AFFECT REG, V0, P46
2017	2018	9.6828	1998	Sheehan DV, 1998, J CLIN PSYCHIAT, V59, P22, DOI 10.4088/JCP.09m05305whi
1996	2002	9.6591	1992	PASINI A, 1992, COMPR PSYCHIAT, V33, P42, DOI 10.1016/0010-440X(92)90078-5
2006	2015	9.6263	2004	Gundel H, 2004, PSYCHOSOM MED, V66, P132, DOI 10.1097/01.PSY.0000097348.45087.96
2011	2015	9.6124	1988	WATSON D, 1988, J PERS SOC PSYCHOL, V54, P1063, DOI 10.1037/0022-3514.54.6.1063
2010	2020	9.6121	2006	Mattila AK, 2006, J PSYCHOSOM RES, V61, P629, DOI 10.1016/j.jpsychores.2006.04.013
2018	2020	9.5515	1997	Taylor G J, 1997, DISORDERS AFFECT REG, V0, P0, DOI 10.1017/CBO9780511526831]
2014	2015	9.4096	2005	Parker PD, 2005, J PERS, V73, P1087, DOI 10.1111/j.1467-6494.2005.00339.x
1993	1997	9.3941	1989	PARKER JDA, 1989, PERS INDIV DIFFER, V10, P599, DOI 10.1016/0191-8869(89)90219-5
2016	2018	9.3399	2015	Brewer R, 2015, NEUROSCI BIOBEHAV R, V56, P348, DOI 10.1016/j.neubiorev.2015.07.006
2007	2017	9.2596	1980	NEMIAH JC, 1970, PSYCHOTHER PSYCHOSOM, V18, P154, DOI 10.1159/000286074
1994	1999	9.175	1992	KAUHANEN J, 1992, J PSYCHOSOM RES, V36, P687, DOI 10.1016/0022-3999(92)90058-A
2005	2008	9.1401	1996	Haviland MG, 1996, J PSYCHOSOM RES, V41, P597, DOI 10.1016/S0022-3999(96)00223-1
2016	2017	9.1078	2009	Honkalampi K, 2009, COMPR PSYCHIAT, V50, P263, DOI 10.1016/j.comppsych.2008.08.007

1995	1997	9.0397	1992	WISE TN, 1992, COMPR PSYCHIAT, V33, P147, DOI 10.1016/0010-440X(92)90023-J
1990	2003	9.0269	1985	PAPCIAK AS, 1985, J HUM STRESS, V11, P135, DOI 10.1080/0097840X.1985.9936750
2018	2020	9.0204	2012	Mehling WE, 2012, PLOS ONE, V7, P0, DOI 10.1371/journal.pone.0048230
2018	2020	9.0204	2017	Bornemann B, 2017, PSYCHOPHYSIOLOGY, V54, P469, DOI 10.1111/psyp.12790
2018	2020	9.0204	2011	Thorberg FA, 2011, EUR PSYCHIAT, V26, P187, DOI 10.1016/j.eurpsy.2010.09.010
2001	2006	8.9762	1996	Lumley MA, 1996, J PSYCHOSOM RES, V41, P519, DOI 10.1016/S0022-3999(96)00227-9
2013	2016	8.9501	2004	Baron-Cohen S, 2004, J AUTISM DEV DISORD, V34, P163, DOI 10.1023/B:JADD.0000022607.19833.00
2012	2015	8.9113	2007	Bailey PE, 2007, PSYCHIAT RES, V150, P13, DOI 10.1016/j.psychres.2006.05.024
2009	2015	8.889	2007	Speranza M, 2007, J PSYCHOSOM RES, V63, P365, DOI 10.1016/j.jpsychores.2007.03.008
1987	2003	8.8791	1985	MARTIN JB, 1985, PSYCHOTHER PSYCHOSOM, V43, P169, DOI 10.1159/000287876
2018	2020	8.8669	2010	Aldao A, 2010, CLIN PSYCHOL REV, V30, P217, DOI 10.1016/j.cpr.2009.11.004
2009	2013	8.8564	1993	CLONINGER CR, 1993, ARCH GEN PSYCHIAT, V50, P975, DOI 10.1001/archpsyc.1993.01820240059008
1981	2000	8.7502	1980	MACLEAN PD, 1949, PSYCHOSOM MED, V11, P338, DOI 10.1097/00006842-194911000-00003
1996	2002	8.706	1993	ZEITLIN SB, 1993, AM J PSYCHIAT, V150, P661
2014	2016	8.5926	2001	Saarijorvi S, 2001, J PSYCHOSOM RES, V51, P729, DOI 10.1016/S0022-3999(01)00257-4

2010	2011	8.546	2007	Luminet O, 2007, J PSYCHOSOM RES, V62, P641, DOI 10.1016/j.jpsychores.2007.01.003
2016	2017	8.5381	2007	Bermond B, 2007, COGNITION EMOTION, V21, P1125, DOI 10.1080/02699930601056989
2018	2020	8.4894	2014	Demartini B, 2014, J NEUROL NEUROSUR PS, V85, P1132, DOI 10.1136/jnnp-2013-307203
2017	2020	8.4597	2007	Jorgensen MM, 2007, PSYCHOTHER PSYCHOSOM, V76, P369, DOI 10.1159/000107565
2010	2014	8.3761	2008	Kugel H, 2008, NEUROSCI LETT, V435, P40, DOI 10.1016/j.neulet.2008.02.005
2016	2018	8.3559	2014	Quattrocki E, 2014, NEUROSCI BIOBEHAV R, V47, P410, DOI 10.1016/j.neubiorev.2014.09.012
2005	2011	8.3272	1999	Zech E, 1999, EUR J PERSONALITY, V13, P511
2011	2013	8.2931	1991	Aiken LS, 1991, MULTIPLE REGRESSION, V0, P0
2014	2015	8.2325	2006	Kessler H, 2006, INT J EAT DISORDER, V39, P245, DOI 10.1002/eat.20228
2008	2011	8.2166	2006	Salminen JK, 2006, J PSYCHOSOM RES, V61, P275, DOI 10.1016/j.jpsychores.2006.01.014
1995	2001	8.1781	1994	BACH M, 1994, COMPR PSYCHIAT, V35, P239, DOI 10.1016/0010-440X(94)90197-X
2015	2020	8.112	1980	FOLSTEIN MF, 1975, J PSYCHIAT RES, V12, P189, DOI 10.1016/0022-3956(75)90026-6
2014	2018	8.1052	2011	Caretti V, 2011, PSYCHIAT RES, V187, P432, DOI 10.1016/j.psychres.2011.02.015
2007	2009	8.0978	1994	HAVILAND MG, 1994, COMPR PSYCHIAT, V35, P124, DOI 10.1016/0010-440X(94)90056-N
1993	1998	8.0711	1990	MCDONALD PW, 1990, PSYCHOSOM MED, V52, P199, DOI 10.1097/00006842-199003000-00007

2006	2010	8.0685	2002	Gunzelmann T, 2002, COMPR PSYCHIAT, V43, P74, DOI 10.1053/comp.2002.29855
2015	2017	8.0573	2013	Gulec MY, 2013, J AFFECT DISORDERS, V146, P137, DOI 10.1016/j.jad.2012.06.033
2008	2014	8.0536	2005	Marchesi C, 2005, PSYCHOTHER PSYCHOSOM, V74, P56, DOI 10.1159/000082028
1982	1986	8.0329	1980	HEIBERG AN, 1980, PSYCHOTHER PSYCHOSOM, V34, P261, DOI 10.1159/000287467
2006	2008	8.0111	2000	Marchesi C, 2000, J PSYCHOSOM RES, V49, P43, DOI 10.1016/S0022-3999(00)00084-2
1984	1988	7.9443	1982	FEIGUINE RJ, 1982, PSYCHOTHER PSYCHOSOM, V37, P185, DOI 10.1159/000287571
2006	2011	7.8428	1999	Jula A, 1999, HYPERTENSION, V33, P1057, DOI 10.1161/01.HYP.33.4.1057
1982	1989	7.8096	1980	FAVA GA, 1980, PSYCHOTHER PSYCHOSOM, V34, P34, DOI 10.1159/000287444
2003	2004	7.7885	1999	Fukunishi I, 1999, J PSYCHOSOM RES, V46, P579, DOI 10.1016/S0022-3999(98)00083-X
2000	2004	7.7295	1997	Friedlander L, 1997, J NERV MENT DIS, V185, P233, DOI 10.1097/00005053-199704000-00003
2018	2020	7.687	1995	LOVIBOND PF, 1995, BEHAV RES THER, V33, P335, DOI 10.1016/0005-7967(94)00075-U
2016	2017	7.663	2006	Rieffe C, 2006, PERS INDIV DIFFER, V40, P123, DOI 10.1016/j.paid.2005.05.013
2017	2020	7.6624	2008	Franz M, 2008, SOC PSYCH PSYCH EPID, V43, P54, DOI 10.1007/s00127-007-0265-1
2016	2018	7.6366	1980	Davis M H, 1980, JSAS CATALOG SELECTE, V10, P85, DOI DOI 10.1037/0022-3514.44.1.113

2000	2006	7.6006	1994	MANN LS, 1994, PSYCHOL REP, V74, P563, DOI 10.2466/pr0.1994.74.2.563
2004	2007	7.5778	2001	Stone LA, 2001, PSYCHOTHER PSYCHOSOM, V70, P92, DOI 10.1159/000056232
2016	2018	7.5484	1980	RADLOFF L S, 1977, APPLIED PSYCHOLOGICAL MEASUREMENT, V1, P385, DOI 10.1177/014662167700100306
2004	2007	7.5461	1990	LANE RD, 1990, J PERS ASSESS, V55, P124, DOI 10.1207/s15327752jpa5501&2_12
2013	2015	7.5446	2002	First MB, 2002, STRUCTURED CLIN INTE, V0, P0
2016	2017	7.5387	2008	Parker JDA, 2008, PSYCHOL ASSESSMENT, V20, P385, DOI 10.1037/a0014262
2017	2018	7.5311	2007	Joukamaa M, 2007, J PSYCHOSOM RES, V63, P373, DOI 10.1016/j.jpsychores.2007.01.018
2017	2020	7.4769	2007	Besharat MA, 2007, PSYCHOL REP, V101, P209, DOI 10.2466/PR0.101.1.209-220
2017	2020	7.447	2013	Samur D, 2013, FRONT PSYCHOL, V4, P0, DOI 10.3389/fpsyg.2013.00861
2016	2017	7.3989	2011	Bird G, 2011, J AUTISM DEV DISORD, V41, P1556, DOI 10.1007/s10803-011-1183-3
2016	2017	7.3989	2013	Aust S, 2013, PSYCHOL TRAUMA-US, V5, P225, DOI 10.1037/a0027314
1988	1990	7.3845	1985	PAULSON JE, 1985, PSYCHOTHER PSYCHOSOM, V44, P57, DOI 10.1159/000287894
2001	2004	7.3501	1997	Fukunishi I, 1997, COMPR PSYCHIAT, V38, P166, DOI 10.1016/S0010-440X(97)90070-5
2017	2020	7.3391	1996	Bressi C, 1996, J PSYCHOSOM RES, V41, P551, DOI 10.1016/S0022-3999(96)00228-0

2000	2004	7.2728	1996	Bach M, 1996, PSYCHOTHER PSYCH MED, V46, P23
1988	1991	7.2505	1987	TAYLOR G, 1987, PSYCHOSOMATIC MED CO, V0, P0
1999	2005	7.2205	1998	Verissimo R, 1998, PSYCHOTHER PSYCHOSOM, V67, P75, DOI 10.1159/000012263
2000	2001	7.1984	1997	Lumley MA, 1997, PSYCHOSOMATICS, V38, P497, DOI 10.1016/S0033-3182(97)71427-0
1999	2010	7.1953	1995	Todarello O, 1995, J PSYCHOSOM RES, V39, P987, DOI 10.1016/0022-3999(95)00506-4
2006	2008	7.1847	1995	LOAS G, 1995, ENCEPHALE, V21, P117
2018	2020	7.1741	2016	Garfinkel SN, 2016, BIOL PSYCHOL, V114, P117, DOI 10.1016/j.biopsycho.2015.12.003
2011	2015	7.1563	1980	OLDFIELD RC, 1971, NEUROPSYCHOLOGIA, V9, P97, DOI 10.1016/0028-3932(71)90067-4
2003	2004	7.139	1999	Roedema TM, 1999, PSYCHOPHYSIOLOGY, V36, P379, DOI 10.1017/S0048577299980290
1992	1999	7.1205	1989	FERNANDEZ A, 1989, PSYCHOTHER PSYCHOSOM, V51, P45, DOI 10.1159/000288133
2008	2011	7.1203	2000	Gohm CL, 2000, PERS SOC PSYCHOL B, V26, P679, DOI 10.1177/0146167200268004
2013	2014	7.1081	2008	De Berardis D, 2008, PROG NEURO-PSYCHOPH, V32, P1982, DOI 10.1016/j.pnpbp.2008.09.022
1995	1997	7.1015	1994	FUKUNISHI I, 1994, PSYCHOL REP, V75, P1371
2011	2012	7.095	2007	van t Wout M, 2007, COMPR PSYCHIAT, V48, P27, DOI 10.1016/j.comppsych.2006.07.003
2004	2005	7.045	1997	Salovey P, 1997, EMOTIONAL DEV EMOTIO, V0, P3, DOI DOI 10.1177/1066480710387486
2004	2005	7.045	2001	Grabe HJ, 2001, PSYCHOTHER PSYCHOSOM, V70, P261, DOI 10.1159/000056264

2011	2020	7.0402	1983	ZIGMOND AS, 1983, ACTA PSYCHIAT SCAND, V67, P361, DOI 10.1111/j.1600-0447.1983.tb09716.x
1980	1990	7.0377	1980	CREMERIUS J, 1977, PSYCHOTHER PSYCHOSOM, V28, P236, DOI 10.1159/000287068
2009	2011	7.0192	2006	Vermeulen N, 2006, COGNITION EMOTION, V20, P64, DOI 10.1080/02699930500304654
1999	2004	6.9958	1998	Kooiman CG, 1998, PSYCHOTHER PSYCHOSOM, V67, P81, DOI 10.1159/000012264
2010	2014	6.979	2006	Meriau K, 2006, NEUROIMAGE, V33, P1016, DOI 10.1016/j.neuroimage.2006.07.031
2009	2015	6.9548	2008	Frewen PA, 2008, J ABNORM PSYCHOL, V117, P171, DOI 10.1037/0021-843X.117.1.171
2014	2017	6.9536	2006	Carano A, 2006, INT J EAT DISORDER, V39, P332, DOI 10.1002/eat.20238
2016	2018	6.8802	2015	Brewer R, 2015, ROY SOC OPEN SCI, V2, P0, DOI 10.1098/rsos.140382
2000	2003	6.8202	1987	COSTA PT, 1987, J PERS, V55, P299, DOI 10.1111/j.1467-6494.1987.tb00438.x
2017	2020	6.7829	2004	Luminet O, 2004, COGNITION EMOTION, V18, P741, DOI 10.1080/02699930341000275
1980	1985	6.7234	1980	Marty P, 1963, INVESTIGATION PSYCHO, V0, P0
2013	2017	6.6934	1980	HAMILTON M, 1960, J NEUROL NEUROSUR PS, V23, P56, DOI 10.1136/jnnp.23.1.56
2016	2018	6.6526	1996	Beck AT, 1996, BECK DEPRESSION INVE, V0, P0
2018	2020	6.635	1988	Cohen J, 1988, STAT POWER ANAL BEHA, V0, P0
1996	2005	6.6065	1994	KAUHANEN J, 1994, PSYCHOSOM MED, V56, P237, DOI 10.1097/00006842-199405000-00010

1985	1989	6.5965	1984	FEDERMAN R, 1984, PSYCHOTHER PSYCHOSOM, V41, P29, DOI 10.1159/000287782
1996	1997	6.5712	1987	SRIRAM TG, 1987, PSYCHOTHER PSYCHOSOM, V47, P11, DOI 10.1159/000287992
1996	1997	6.5712	1992	WILKINSON L, 1992, SYSTAT STAT VERSION, V0, P0
2004	2008	6.5476	2000	Grabe HJ, 2000, PSYCHOTHER PSYCHOSOM, V69, P128, DOI 10.1159/000012380
2017	2020	6.545	2008	de Timary P, 2008, PSYCHIAT RES, V157, P105, DOI 10.1016/j.psychres.2006.12.008
1986	1991	6.4875	1984	COOPER DE, 1984, PSYCHOTHER PSYCHOSOM, V41, P20, DOI 10.1159/000287780
2016	2018	6.4702	2001	Kokkonen P, 2001, COMPR PSYCHIAT, V42, P471, DOI 10.1053/comp.2001.27892
2012	2013	6.457	1998	Parker JDA, 1998, COMPR PSYCHIAT, V39, P91, DOI 10.1016/S0010-440X(98)90084-0
2011	2017	6.4567	2003	Guilbaud O, 2003, BIOMED PHARMACOTHER, V57, P292, DOI 10.1016/S0753-3322(03)00085-4
2004	2007	6.4113	2001	Porcelli P, 2001, PSYCHOTHER PSYCHOSOM, V70, P184, DOI 10.1159/000056251
1991	1995	6.3991	1988	HAVILAND MG, 1988, PSYCHOTHER PSYCHOSOM, V49, P37, DOI 10.1159/000288065
2007	2010	6.386	2005	Lumley MA, 2005, EMOTION, V5, P329, DOI 10.1037/1528-3542.5.3.329
2002	2004	6.3363	1997	Rastam M, 1997, ACTA PSYCHIAT SCAND, V95, P385, DOI 10.1111/j.1600-0447.1997.tb09650.x
2007	2008	6.2277	1996	Linden W, 1996, J PSYCHOSOM RES, V41, P575, DOI 10.1016/S0022-3999(96)00229-2
2013	2017	6.2165	2010	Parling T, 2010, EAT BEHAV, V11, P205, DOI 10.1016/j.eatbeh.2010.04.001

2010	2013	6.2076	2008	Helmes E, 2008, J CLIN PSYCHOL, V64, P318, DOI 10.1002/jclp.20461
2001	2004	6.1004	1998	Kooiman CG, 1998, COMPR PSYCHIAT, V39, P152, DOI 10.1016/S0010-440X(98)90075-X
2001	2004	6.1004	1988	LIPOWSKI ZJ, 1988, AM J PSYCHIAT, V145, P1358
2016	2020	6.091	2013	Shishido H, 2013, ADDICT BEHAV, V38, P2014, DOI 10.1016/j.addbeh.2012.12.014
2006	2009	6.0398	2004	Eizaguirre AE, 2004, PERS INDIV DIFFER, V36, P321, DOI 10.1016/S0191-8869(03)00099-0
1993	1999	6.0373	1989	HORTON PC, 1989, PSYCHOTHER PSYCHOSOM, V51, P91, DOI 10.1159/000288141
1992	1994	5.9725	1991	PARKER JDA, 1991, PSYCHOSOMATICS, V32, P196, DOI 10.1016/S0033-3182(91)72092-6
1983	1995	5.9335	1980	ALEXANDER F, 1950, PSYCHOSOMATIC MED, V0, P0
1997	1999	5.9141	1995	FUKUNISHI I, 1995, PSYCHOL REP, V76, P1299, DOI 10.2466/pr0.1995.76.3c.1299
1998	2000	5.8999	1985	Costa P T, 1985, NEO PERSONALITY INVE, V0, P0
1996	1998	5.8163	1993	PRINCE JD, 1993, J RES PERS, V27, P15, DOI 10.1006/jrpe.1993.1002
2006	2007	5.7229	1984	Lazarus R S, 1984, STRESS APPRAISAL COP, V0, P0
2005	2008	5.7107	2004	Kooiman CG, 2004, PSYCHOTHER PSYCHOSOM, V73, P107, DOI 10.1159/000075542
2002	2004	5.7024	1993	PARKER JDA, 1993, NEW TRENDS EXPER CLI, V9, P9
2015	2018	5.6929	2004	BATEMAN A, 2004, PSYCHOTHERAPY BORDER, V0, P0
1996	2003	5.612	1987	Kirmayer LJ, 1987, TRANSCULT PSYCHIATR, V24, P119

2004	2012	5.5032	1998	Schutte NS, 1998, PERS INDIV DIFFER, V25, P167, DOI 10.1016/S0191-8869(98)00001-4
1990	1996	5.5003	1985	CATCHLOVE RFH, 1985, J NERV MENT DIS, V173, P246, DOI 10.1097/00005053-198504000-00007
1983	1985	5.4357	1982	DEMERSDESROSIERS L, 1982, PSYCHOTHER PSYCHOSOM, V38, P103, DOI 10.1159/000287619
1997	2002	5.4237	1989	WATSON D, 1989, PSYCHOL REV, V96, P234, DOI 10.1037/0033-295X.96.2.234
1988	1989	5.4201	1988	TAYLOR GJ, 1988, PSYCHOSOM MED, V50, P205
1993	1994	5.392	1988	SIFNEOS PE, 1988, PSYCHIAT CLIN N AM, V11, P287
2007	2009	5.3758	2001	Cedro A, 2001, PSYCHOL REP, V89, P95, DOI 10.2466/pr0.2001.89.1.95
2010	2015	5.3522	2008	McRae K, 2008, NEUROIMAGE, V41, P648, DOI 10.1016/j.neuroimage.2008.02.030
2010	2015	5.3522	1980	Ekman P, 1976, PICTURES FACIAL AFFE, V0, P0
2012	2013	5.3506	1995	FAVA GA, 1995, PSYCHOTHER PSYCHOSOM, V63, P1, DOI 10.1159/000288931
1997	1998	5.3491	1980	Marty P, 1963, REV FRANC PSYCHANAL, V27, P345
1992	1994	5.3086	1989	NORTON NC, 1989, J PERS ASSESS, V53, P621, DOI 10.1207/s15327752jpa5303_18
1997	1999	5.2567	1994	WISE TN, 1994, J PSYCHOSOM RES, V38, P515, DOI 10.1016/0022-3999(94)90048-5
1987	1991	5.2508	1982	BLUMER D, 1982, J NERV MENT DIS, V170, P381, DOI 10.1097/00005053-198207000-00001
1999	2001	5.1419	1993	KAUHANEN J, 1993, COMPR PSYCHIAT, V34, P330, DOI 10.1016/0010-440X(93)90019-Z
1994	2007	5.1279	1980	CROWNE DP, 1960, J CONSULT PSYCHOL, V24, P349, DOI 10.1037/h0047358

1991	1995	5.1187	1988	LOISELLE CG, 1988, PSYCHOTHER PSYCHOSOM, V50, P109, DOI 10.1159/000288108
2005	2010	5.1016	2003	Muller J, 2003, J PSYCHOSOM RES, V55, P561, DOI 10.1016/S0022-3999(03)00033-3
1992	1996	5.0368	1991	BAGBY RM, 1991, J PERS ASSESS, V56, P238, DOI 10.1207/s15327752jpa5602_5
2003	2011	5.0151	1997	Taylor GJ, 1997, DISORDERS AFFECT REG, V0, P26, DOI DOI 10.1017/CBO9780511526831.005
1999	2005	4.9977	1996	Porcelli P, 1996, J PSYCHOSOM RES, V41, P569, DOI 10.1016/S0022-3999(96)00221-8
1999	2003	4.8669	1997	Infrasca R, 1997, PSYCHOTHER PSYCHOSOM, V66, P276, DOI 10.1159/000289147
2000	2004	4.7574	1986	BERNSTEIN EM, 1986, J NERV MENT DIS, V174, P727, DOI 10.1097/00005053-198612000-00004
1985	1986	4.747	1980	HOPPE KD, 1977, PSYCHOANAL QUART, V46, P220, DOI 10.1080/21674086.1977.11926798
1985	1987	4.7283	1983	COHEN KR, 1983, PSYCHOTHER PSYCHOSOM, V39, P77, DOI 10.1159/000287725
1989	1998	4.6427	1986	AHRENS S, 1986, AM J PSYCHOTHER, V40, P430
1984	1988	4.633	1981	BROWN EL, 1981, PSYCHOTHER PSYCHOSOM, V36, P116, DOI 10.1159/000287534
2007	2013	4.6321	2004	De Gucht V, 2004, PERS INDIV DIFFER, V36, P1655, DOI 10.1016/j.paid.2003.06.012
1998	2000	4.5883	1996	Bach M, 1996, PSYCHOTHER PSYCHOSOM, V65, P150, DOI 10.1159/000289067
2008	2015	4.5374	1994	Damasio A R, 1994, DESCARTES ERROR EMOT, V0, P0
2004	2013	4.4711	1995	WEHMER F, 1995, J NERV MENT DIS, V183, P351, DOI 10.1097/00005053-199506000-00001

2006	2012	4.461	1980	Bruch H, 1973, EATING DISORDERS OBE, V0, P0
2006	2012	4.461	2004	Kucharska-Pietura K, 2004, INT J EAT DISORDER, V35, P42, DOI 10.1002/eat.10219
2006	2013	4.4285	2001	Vorst HCM, 2001, PERS INDIV DIFFER, V30, P413, DOI 10.1016/S0191-8869(00)00033-7
2008	2014	4.4274	2004	Franz M, 2004, AM J PSYCHIAT, V161, P728, DOI 10.1176/appi.ajp.161.4.728
2016	2017	4.2923	2006	Bermond Bob, 2006, COGN NEUROPSYCHIATRY, V11, P332, DOI 10.1080/13546800500368607
2005	2010	4.2006	1993	PARKER JDA, 1993, AM J PSYCHIAT, V150, P1105
2017	2018	4.1885	2004	Waller E, 2004, J PSYCHOSOM RES, V57, P239, DOI 10.1016/S0022-3999(03)00613-5
2005	2006	4.1066	1987	LANE RD, 1987, AM J PSYCHIAT, V144, P133
2016	2017	4.1038	2007	Vanheule S, 2007, J CLIN PSYCHOL, V63, P109, DOI 10.1002/jclp.20324
1987	1988	4.0845	1986	TENHOUTEN WD, 1986, AM J PSYCHIAT, V143, P312
2002	2004	4.0586	1994	BAGBY RM, 1994, J PSYCHOSOM RES, V38, P33, DOI 10.1016/0022-3999(94)90006-X
1989	1991	4.0134	1980	Cattell RB, 1978, SCI USE FACTOR ANAL, V0, P0
1994	2001	4.0059	1992	FUKUNISHI I, 1992, PSYCHOTHER PSYCHOSOM, V57, P50, DOI 10.1159/000288573
1981	1986	4.0035	1980	SCHNEIDER PB, 1977, PSYCHOTHER PSYCHOSOM, V28, P36, DOI 10.1159/000287042
1984	1988	3.9709	1980	Krystal H, 1970, DRUG DEPENDENCE, V0, P0
1988	1991	3.9537	1980	BECK AT, 1979, COGNITIVE THERAPY DE, V0, P0
2009	2010	3.9078	2000	Honkalampi K, 2000, J PSYCHOSOM RES, V48, P99, DOI 10.1016/S0022-3999(99)00083-5

2014	2017	3.7954	2005	Spitzer C, 2005, PSYCHOTHER PSYCHOSOM, V74, P240, DOI 10.1159/000085148

Supplementary Table 3 List of the documents with highest centrality or sigma, estimated via document co-citation analysis (DCA).

Centrality	Reference	Cluster
0.14	Sifneos, 1973	1
0.13	Freyberger, 1977	1
0.11	Taylor, 1984	1
0.10	Nemiah et al., 1976	0
0.09	Taylor et al., 1991	2
0.08	Lane & Schwartz, 1987	3
0.08	Hamilton, 1960	4
0.07	Taylor et al., 1985	2
0.07	Honkalampi et al., 2000	0
0.07	Apfel & Sifneos, 1979	1
0.06	Salminen et al., 1999	0
0.06	Bagby et al., 2006	0
0.06	Krystal, 1979	1
0.06	Sifneos, 1972	1
**Sigma (∑)**	**Reference**	**Cluster**
693.31	Taylor, 1984	1
151.25	Taylor et al., 1985	2
76.13	Freyberger, 1977	1
38.48	Apfel & Sifneos, 1979	1
10.52	Taylor et al., 1991	2

8.21	Krystal, 1979	1
7.53	Sifneos, 1972	1
7.29	American Psychiatric Association, 1994	0
6.34	Taylor et al., 1988	2
5.29	Lesser, 1981	1
4.38	Kleiger & Kinsman, 1980	1
4.17	Nemiah & Sifneos, 1970	2
3.56	Taylor & Bagby, 2004	0
3.41	Bagby et al., 1986	2
3.01	Hendryx et al., 1991	2

Supplementary Table 4 Journals with a citation burst of at least two years computed via journal co-citation analysis (JCA). Journals are ordered by burst strength.

Journal	Burst Strength	Begin	End	Duration
PSYCHOTHER PSYCHOSOM	**164.51**	1980	2006	26
PSYCHOSOM MED	**138.78**	1980	2006	26
AM J PSYCHIAT	**125.12**	1985	2006	21
FRONT PSYCHOL	**88.00**	2016	2020	4
DISORDERS AFFECT REG	**87.49**	1998	2010	12
PLOS ONE	**74.82**	2015	2020	5
MODERN TRENDS PSYCHO	**73.19**	1980	2001	21
J NERV MENT DIS	**65.90**	1981	2004	23
AM J PSYCHOTHER	**58.20**	1981	2002	21
PSYCHOSOMATICS	**56.71**	1992	2005	13
SHORT TERM PSYCHOTHE	**51.76**	1981	2001	20
BRIT J PSYCHIAT	**47.05**	1993	2006	13
PSYCHIAT CLIN N AM	**46.64**	1988	2001	13
PSYCHOL REP	**46.48**	1995	2007	12
BRIT J MED PSYCHOL	**40.12**	1990	2007	17
NEW ENGL J MED	**35.91**	1985	2008	23
GEN HOSP PSYCHIAT	**35.26**	1981	2001	20
INT J PSYCHOANAL PSYCHOTHER	**34.19**	1984	2004	20
EUR J PERSONALITY	**33.69**	1995	2008	13
HDB EMOTIONAL INTELL	**33.46**	2002	2011	9
NEW TRENDS EXPT CLIN	**31.10**	1995	2003	8
FRONT PSYCHIATRY	**31.05**	2017	2020	3
THESIS	**30.27**	2016	2020	4
SOC COGN AFFECT NEUR	**29.04**	2014	2020	6
SCI REP-UK	**29.03**	2017	2020	3
J HUM STRESS	**28.82**	1986	2004	18
FRONT HUM NEUROSCI	**27.74**	2015	2020	5
NEUROSCI BIOBEHAV R	**27.12**	2016	2020	4
ACTA PSYCHIAT SCAND	**25.69**	1996	2004	8
TRANSL PSYCHIAT	**25.36**	2015	2020	5
AM J MED	**24.03**	1982	2004	22
PROCD SOC BEHV	**21.61**	2016	2020	4
AUTISM RES	**21.36**	2016	2020	4
NEUROPSYCH DIS TREAT	**21.22**	2016	2020	4
ARCH GEN PSYCHIAT	**20.39**	1980	2005	25
INTRO MEDIATION MODE	**19.81**	2016	2020	4
PSYCHIAT INVEST	**19.64**	2016	2020	4
CAN J PSYCHIAT	**19.56**	2001	2007	6

ROY SOC OPEN SCI	**19.50**	2016	2020	4
BMC PSYCHIATRY	**19.38**	2017	2020	3
BEHAV MED	**19.17**	1995	2007	12
PSYCHOTHER PSYCH MED	**19.00**	2000	2010	10
IMAGINATION COGNITIO	**18.99**	1999	2007	8
EMOTION DISCLOSURE H	**18.26**	1998	2011	13
AM J EPIDEMIOL	**18.24**	1999	2013	14
J PSYCHOPATHOL BEHAV	**18.22**	2017	2020	3
JAMA PSYCHIAT	**18.05**	2018	2020	2
PERSONAL DISORD	**17.95**	2016	2020	4
INT J PSYCHIAT MED	**17.78**	1982	2001	19
INT REV PSYCHOANAL	**17.48**	1980	1991	11
INTELLIGENCE	**17.11**	2002	2011	9
AM J GERIAT PSYCHIAT	**16.89**	2013	2017	4
ASIAN J PSYCHIATR	**16.65**	2018	2020	2
DIAGNOSTIC STAT MANU	**16.65**	2018	2020	2
J EXP PSYCHOL GEN	**16.58**	2018	2020	2
PSYCHOANAL QUART	**16.55**	1983	1997	14
J TRAUMA DISSOCIATIO	**16.35**	2015	2018	3
FRONT BEHAV NEUROSCI	**16.09**	2016	2020	4
J PSYCHIAT RES	**16.05**	1985	2000	15
MINDFULNESS	**15.73**	2018	2020	2
DEV COGN NEUROS-NETH	**15.73**	2018	2020	2
BRAIN STRUCT FUNCT	**15.65**	2015	2020	5
COMPUT HUM BEHAV	**15.61**	2018	2020	2
CLIN PSYCHOL SCI	**15.61**	2018	2020	2
PSYCHOL ADDICT BEHAV	**15.01**	2017	2020	3
CURR PSYCHIAT REP	**14.98**	2017	2020	3
CLIN NEUROPSYCHIATR	**14.81**	2017	2020	3
REVISED NEO PERSONAL	**14.65**	2003	2008	5
APPETITE	**14.30**	2015	2020	5
BRIT MED J	**14.20**	1992	2003	11
EMOTIONAL INTELLIGEN	**14.12**	2000	2007	7
EMOT REV	**14.12**	2018	2020	2
NEW TRENDS EXPER CLI	**14.11**	1998	2005	7
PSYCHOTHERAPY	**13.94**	2017	2018	1
MOL AUTISM	**13.93**	2016	2020	4
BMC PUBLIC HEALTH	**13.87**	2018	2020	2
HYPERTENSION	**13.85**	2005	2011	6
PSYCHOL TRAUMA-US	**13.83**	2014	2020	6
PSYCHOANAL PSYCHOL	**13.63**	2016	2020	4
NEUROREPORT	**13.57**	2006	2014	8
MOL PSYCHIATR	**13.42**	2018	2020	2

EAT WEIGHT DISORD-ST	**13.36**	2018	2020	2
PSYCHOL INQ	**13.35**	2017	2020	3
DIAGN STAT MAN MENT	**13.34**	2001	2004	3
SUBST USE MISUSE	**13.10**	2018	2020	2
ALCOHOL ALCOHOLISM	**13.03**	2009	2013	4
PSYCHIATRY	**13.00**	2009	2017	8
PSYCHIAT QUART	**12.90**	2017	2020	3
J EAT DISORD	**12.80**	2017	2020	3
AM J PSYCHOL	**12.79**	2017	2020	3
EUR J NEUROSCI	**12.75**	2012	2016	4
CIRCULATION	**12.65**	1995	2010	15
PHYSIOL BEHAV	**12.61**	2017	2020	3
J CLIN EPIDEMIOL	**12.52**	2017	2020	3
EUR J PAIN	**12.48**	2013	2016	3
BEHAV RES METHODS	**12.34**	2014	2020	6
PSYCHOPATHOLOGY	**12.27**	2005	2008	3
CURR BIOL	**12.22**	2013	2018	5
HARVARD REV PSYCHIAT	**12.19**	2015	2020	5
ANNU REV CLIN PSYCHO	**12.19**	2016	2020	4
J ADOLESCENT HEALTH	**12.16**	2016	2018	2
RES AUTISM SPECT DIS	**12.06**	2016	2020	4
AUTISM	**11.99**	2016	2020	4
ANN INTERN MED	**11.96**	1991	2008	17
ARCH INTERN MED	**11.93**	1991	2007	16
PSYCHOSOMATIC MED TH	**11.91**	1985	1994	9
BEHAV BRAIN RES	**11.88**	2014	2020	6
J CONSULT CLIN PSYCH	**11.85**	2002	2005	3
HEALTH PSYCHOL	**11.84**	1996	2007	11
PEDIATRICS	**11.82**	2017	2020	3
EMOTIONAL DEV EMOTIO	**11.69**	2000	2006	6
COMPR PSYCHIAT	**11.68**	2001	2003	2
AM J CARDIOL	**11.67**	1995	2004	9
WORLD PSYCHIATRY	**11.65**	2017	2018	1
BRAIN RES	**11.62**	2013	2016	3
J BEHAV ADDICT	**11.56**	2018	2020	2
PSYCHOSOMATIC MED CO	**11.49**	1988	2001	13
J NEUROPSYCH CLIN N	**11.48**	2015	2020	5
INVESTIGATION PSYCHO	**11.42**	1980	1997	17
BIOPSYCHOSOC MED	**11.28**	2014	2018	4
NEUROPSYCHOBIOLOGY	**11.20**	2013	2016	3
NEUROSCI LETT	**11.19**	2012	2016	4
SOC NEUROSCI-UK	**11.17**	2013	2020	7
NEUROSCIENCE	**11.08**	2015	2016	1
J HEALTH PSYCHOL	**11.08**	2016	2020	4

ATTACHMENT LOSS	**11.06**	2017	2020	3
CHILD PSYCHIAT HUM D	**11.03**	2017	2018	1
MANUAL BECK DEPRESSI	**11.02**	2013	2018	5
PERSPECT PSYCHOL SCI	**11.00**	2013	2020	7
PSYCHIAT RES-NEUROIM	**10.92**	2012	2015	3
PSYCHOTHER RES	**10.88**	2017	2020	3
ADDICT RES THEORY	**10.87**	2018	2020	2
ARCH SUICIDE RES	**10.86**	2016	2018	2
ANN BEHAV MED	**10.85**	2005	2009	4
EMOTION REGULATION C	**10.72**	2012	2015	3
MOVEMENT DISORD	**10.72**	2015	2020	5
GUT	**10.71**	2003	2007	4
PERS SOC PSYCHOL REV	**10.69**	2017	2020	3
USING MULTIVARIATE S	**10.64**	2015	2018	3
MPLUS USERS GUIDE	**10.64**	2018	2020	2
AM PSYCHOL	**10.63**	1989	2004	15
J NEUROL	**10.61**	2014	2017	3
KLIN PSIKOFARMAKOL B	**10.47**	2017	2020	3
MED CARE	**10.45**	2008	2013	5
INT J MENT HEALTH AD	**10.30**	2017	2020	3
J PSYCHOL	**10.27**	2017	2020	3
PROG BRAIN RES	**10.21**	2014	2017	3
DEVELOPMENTAL SCI	**10.17**	2018	2020	2
PSYCHON B REV	**10.17**	2018	2020	2
ACTA NEUROPSYCHIATR	**10.10**	2014	2015	1
PSYCHOMETRIC THEORY	**10.05**	1988	2002	14
CURR OPIN NEUROBIOL	**9.99**	2013	2015	2
ANN NEUROL	**9.92**	2015	2017	2
INT REV PSYCHIATR	**9.91**	2013	2016	3
PROG NEURO-PSYCHOPH	**9.81**	2013	2016	3
J FAM PSYCHOL	**9.80**	2014	2017	3
STATE TRAIT ANXIETY	**9.73**	2010	2014	4
PSYCHOANAL INQ	**9.70**	2017	2018	1
NEUROPSYCHOL REV	**9.65**	2013	2016	3
JAMA-J AM MED ASSOC	**9.63**	1997	2003	6
SPAN J PSYCHOL	**9.62**	2017	2018	1
J PSYCHIATR NEUROSCI	**9.62**	2012	2015	3
CEREB CORTEX	**9.60**	2014	2016	2
ANNU PSYCHOANAL	**9.54**	1982	2005	23
PSICOTHEMA	**9.50**	2011	2013	2
BEHAV COGN NEUROSCI REV	**9.46**	2008	2015	7
ARCH NEUROL-CHICAGO	**9.45**	2007	2013	6
J CLIN PSYCHIAT	**9.43**	1994	2002	8

J SOC CLIN PSYCHOL	**9.35**	2011	2013	2
NEUROCASE	**9.34**	2013	2018	5
PERSONAL MENT HEALTH	**9.28**	2016	2020	4
EPILEPSY BEHAV	**9.26**	2018	2020	2
J ABNORM CHILD PSYCH	**9.21**	2017	2020	3
COMPREHENSIVE TXB PS	**9.16**	1995	1999	4
INT J PSYCHOL	**9.15**	2017	2020	3
CORTEX	**9.03**	2015	2018	3
TRENDS COGN SCI	**9.00**	2015	2016	1
J AM ACAD PSYCHOAN	**8.87**	1986	1998	12
ATTACH HUM DEV	**8.85**	2018	2020	2
CYBERPSYCH BEH SOC N	**8.79**	2018	2020	2
BRAIN INJURY	**8.73**	2011	2016	5
JSAS CATALOG SELECTE	**8.69**	2016	2018	2
PSYCHOSOMATIC MED	**8.50**	1983	1995	12
REV FRANC PSYCHANAL	**8.46**	1988	2001	13
J NEUROPHYSIOL	**8.42**	2014	2015	1
AFFECT REGULATION ME	**8.38**	2016	2018	2
SEIZURE-EUR J EPILEP	**8.35**	2015	2018	3
AM J GASTROENTEROL	**8.32**	2003	2005	2
ANN GEN PSYCHIATR	**8.31**	2017	2018	1
PROG NEUROBIOL	**8.25**	2016	2018	2
TRENDS NEUROSCI	**8.09**	2015	2016	1
MULTIPLE REGRESSION	**8.00**	2011	2013	2
PSYCHOTHERAPY AND PSYCHOSOMATICS	**7.94**	1983	2000	17
PSYCHOPHARMACOLOGY	**7.91**	2017	2020	3
MOTIV EMOTION	**7.88**	2018	2020	2
COCHRANE DB SYST REV	**7.87**	2014	2016	2
SCHIZOPHR RES	**7.85**	2011	2012	1
PSYCHOL REC	**7.83**	2014	2016	2
MASSIVE PSYCHIC TRAU	**7.81**	1987	1996	9
AM J DRUG ALCOHOL AB	**7.77**	2017	2020	3
J ADV NURS	**7.75**	2013	2014	1
APPL MULTIPLE REGRES	**7.71**	2006	2009	3
BEHAV COGN PSYCHOTH	**7.63**	2018	2020	2
J INT NEUROPSYCH SOC	**7.58**	2010	2015	5
BRAIN BEHAV IMMUN	**7.56**	2016	2020	4
ANNU REV NEUROSCI	**7.51**	2013	2016	3
J PAIN	**7.47**	2018	2020	2
BEHAV MODIF	**7.44**	2010	2014	4
J GEN INTERN MED	**7.42**	2017	2020	3
J HEALTH SOC BEHAV	**7.38**	2017	2018	1
PATIENT EDUC COUNS	**7.37**	2006	2011	5

NEUROPSYCHOLOGY	**7.34**	2010	2011	1
J COMP NEUROL	**7.31**	2016	2018	2
J PEDIATR PSYCHOL	**7.30**	2008	2010	2
AGGRESS VIOLENT BEH	**7.25**	2017	2020	3
BEHAV SCI LAW	**7.25**	2017	2020	3
BECK DEPRESSION INVE	**7.21**	2010	2013	3
ACTA NEUROL SCAND	**7.20**	2011	2017	6
J CLIN CHILD ADOLESC	**7.17**	2015	2017	2
EAT DISORD	**7.15**	2012	2015	3
J CONSULT PSYCHOL	**7.14**	2007	2008	1
BEHAV RES METH INS C	**7.10**	2014	2015	1
SOC SCI MED	**7.06**	1998	2007	9
PSYCHOL HEALTH	**7.06**	2005	2011	6
COGN AFFECT BEHAV NE	**7.04**	2015	2020	5
NEO PERSONALITY INVE	**6.97**	1993	2000	7
SEX ROLES	**6.96**	2013	2015	2
TRANSCULT PSYCHIATR	**6.96**	1989	2003	14
CLIN RHEUMATOL	**6.94**	2018	2020	2
ARCH CLIN NEUROPSYCH	**6.91**	2015	2020	5
ARTHRITIS RHEUM	**6.89**	2002	2005	3
SYSTAT STAT VERSION	**6.76**	1996	1997	1
EPILEPSIA	**6.75**	2013	2015	2
COGNITIVE BRAIN RES	**6.70**	2012	2015	3
ALCOHOL CLIN EXP RES	**6.68**	2018	2020	2
J CLIN PSYCHOL	**6.68**	2002	2003	1
7TH P EUR C PSYCH RE	**6.68**	1980	1992	12
NORD J PSYCHIAT	**6.61**	2003	2008	5
PSYCHOL PHYSICAL SYM	**6.59**	1991	1996	5
J SOC PERS RELAT	**6.58**	2014	2017	3
J BEHAV THER EXP PSY	**6.56**	2011	2015	4
J PERS ASSESS	**6.53**	2000	2003	3
DISSOCIATION	**6.44**	2016	2018	2
BIOMED PHARMACOTHER	**6.40**	2011	2013	2
TRANSCULT PSYCHIATRY	**6.35**	1988	2003	15
PHILOS T R SOC B	**6.35**	2013	2020	7
J RES PERS	**6.34**	1998	2000	2
PSYCHOL REV	**6.32**	1996	2003	7
NEURON	**6.22**	2013	2015	2
COGN BEHAV NEUROL	**6.22**	2013	2017	4
J STUD ALCOHOL	**6.19**	1988	2012	24
J APPL PSYCHOL	**6.18**	2010	2013	3
TOPICS PSYCHOSOMATIC	**6.16**	1980	1987	7
NAT REV NEUROSCI	**6.12**	2015	2016	1

J INTERPERS VIOLENCE	**6.04**	2015	2016	1
NAT NEUROSCI	**6.00**	2014	2016	2
HDB EMOTION REGULATI	**5.98**	2017	2020	3
REPRESSION DISSOCIAT	**5.94**	1994	2006	12
J CONT ED PSYCHIATRY	**5.87**	1988	1996	8
COGNITIVE THERAPY DE	**5.87**	1988	1996	8
B MENNINGER CLIN	**5.75**	1997	2007	10
BRAIN RES REV	**5.69**	2006	2011	5
EUR ARCH PSY CLIN N	**5.67**	2010	2012	2
STRUCTURED CLIN INTE	**5.67**	1994	1997	3
LANCET NEUROL	**5.64**	2017	2020	3
PSYCHOMETRIKA	**5.61**	2009	2013	4
STRESS MEDICINE	**5.60**	2000	2004	4
EATING DISORDER INVE	**5.58**	2006	2012	6
APPLIED PSYCHOLOGICAL MEASUREMENT	**5.57**	2016	2018	2
PSYCHIAT CLIN NEUROS	**5.51**	2010	2015	5
SCHIZOPHRENIA BULL	**5.45**	2011	2012	1
NERVENARZT	**5.44**	2004	2014	10
CURR OPIN PSYCHIATR	**5.43**	2011	2015	4
PERCEPT MOTOR SKILL	**5.42**	2005	2007	2
COGNITION	**5.36**	2015	2016	1
QUAL LIFE RES	**5.33**	2018	2020	2
AM J PUBLIC HEALTH	**5.29**	2008	2009	1
AM HEART J	**5.28**	1996	1999	3
PERS SOC PSYCHOL B	**5.28**	2007	2011	4
STRESS RESPONSE SYND	**5.25**	2000	2002	2
BRAIN	**5.22**	2015	2016	1
INT J GROUP PSYCHOTH	**5.21**	1991	1997	6
STANDARD EDITION	**5.21**	1991	1997	6
MINNESOTA MULTIPHASI	**5.20**	1996	2000	4
J ANXIETY DISORD	**5.19**	2011	2013	2
HDB EMOTIONS	**5.18**	2012	2015	3
EUR CHILD ADOLES PSY	**5.14**	2006	2010	4
EATING DISORDERS OBE	**5.12**	2006	2012	6
ATTACHMENT THEORY CL	**5.12**	2003	2005	2
BRIT J PSYCHOL	**5.06**	2007	2009	2
EDUC PSYCHOL MEAS	**5.04**	2010	2011	1
TURK PSIKIYATR DERG	**5.02**	2017	2018	1
INT J PSYCHOANAL	**5.01**	2002	2005	3
CLIN J PAIN	**5.00**	2013	2016	3
SCIENCE	**4.92**	2007	2008	1
J CLIN EXP NEUROPSYC	**4.89**	2017	2020	3
SOC PSYCH PSYCH EPID	**4.87**	2013	2014	1

AM J ADDICTION	**4.76**	2008	2012	4
PSYCHOL METHODS	**4.62**	2007	2012	5
DIGEST DIS SCI	**4.57**	2001	2003	2
ANN MED-PSYCHOL	**4.55**	2000	2006	6
PSYCHOANAL STUDY CHILD	**4.51**	1991	1998	7
J ABNORM SOC PSYCH	**4.49**	1998	2002	4
J COGNITIVE NEUROSCI	**4.47**	2005	2006	1
NONEXPRESSION EMOTIO	**4.44**	1999	2003	4
J COUNS PSYCHOL	**4.42**	2002	2009	7
ADV PSYCHOSOM MED	**4.41**	1983	1993	10
EMOTIONS	**4.34**	1998	2007	9
OBES RES	**4.27**	2012	2014	2
J NONVERBAL BEHAV	**4.27**	2011	2013	2
J NEUROSCI	**4.23**	2014	2016	2
J AM PSYCHOANAL ASS	**4.18**	2016	2018	2
AM J ORTHOPSYCHIAT	**4.18**	2014	2018	4
J BEHAV MED	**4.15**	1988	1995	7
CLIN PSYCHOL-SCI PR	**4.15**	2008	2009	1
DRUG DEPENDENCE	**4.11**	1984	1988	4
SOC BEHAV PERSONAL	**4.10**	2002	2006	4
SCI USE FACTOR ANAL	**4.10**	1989	1991	2
BRIT J HEALTH PSYCH	**4.09**	2017	2020	3
THEORY PSYCHOSOMATIC	**4.08**	1980	1989	9
EATING DISORDERS	**4.05**	1996	1997	1
50 ANN M AM PSYCH SO	**4.05**	1996	1997	1
AUST NZ J PSYCHIAT	**4.01**	2012	2013	1
GASTROENTEROLOGY	**4.00**	2013	2016	3
DIABETES CARE	**3.93**	1997	2006	9
EUR J PSYCHOL ASSESS	**3.87**	2009	2012	3
MULTIVAR BEHAV RES	**3.86**	2007	2009	2
PSYCHOL MEN MASCULIN	**3.82**	2012	2015	3
REV FRANCAISE PSYC S	**3.79**	2005	2007	2
ADV PERS ASSESS	**3.74**	1996	2002	6

Supplementary Table 5. List of citing documents. For each cluster are reported the top 20 citing publications for Global Citation Score (GCS). Coverage represents the number of references within the cluster that are cited by each document.

Cluter ID	Bibliography	GCS	Coverage
0	Reise, Steven P (2013) Scoring and modeling psychological measures in the presence of multidimensionality. JOURNAL OF PERSONALITY ASSESSMENT, V95, P12 DOI 10.1080/00223891.2012.725437	292	3
0	Herbert, Beate M (2011) On the relationship between interoceptive awareness and alexithymia: is interoceptive awareness related to emotional awareness?. JOURNAL OF PERSONALITY, V79, P27 DOI 10.1111/j.1467-6494.2011.00717.x	181	12
0	Berthoz, S (2005) The validity of using self-reports to assess emotion regulation abilities in adults with autism spectrum disorder. EUROPEAN PSYCHIATRY DOI 10.1016/j.eurpsy.2004.06.013	180	3
0	Vellante, Marcello (2013) The "reading the mind in the eyes" test: systematic review of psychometric properties and a validation study in italy. COGNITIVE NEUROPSYCHIATRY, V18, P29 DOI 10.1080/13546805.2012.721728	154	5
0	Cook, Richard (2013) Alexithymia, not autism, predicts poor recognition of emotional facial expressions. PSYCHOLOGICAL SCIENCE, V24, P10 DOI 10.1177/0956797612463582	127	3
0	Luminet, O (1999) Relation between alexithymia and the five-factor model of personality: a facet-level analysis. JOURNAL OF PERSONALITY ASSESSMENT, V73, P14 DOI 10.1207/S15327752JPA7303_4	116	10
0	Spitzer, C (2005) Alexithymia and interpersonal problems. PSYCHOTHERAPY AND PSYCHOSOMATICS DOI 10.1159/000085148	109	3
0	Grynberg, Delphine (2010) Alexithymia in the interpersonal domain: a general deficit of empathy?. PERSONALITY AND INDIVIDUAL DIFFERENCES DOI 10.1016/j.paid.2010.07.013	103	6
0	Picardi, A (2005) Stability of alexithymia and its relationships with the 'big five' factors, temperament, character, and attachment style. PSYCHOTHERAPY AND PSYCHOSOMATICS DOI 10.1159/000087785	97	3
0	van der Velde, Jorien (2013) Neural correlates of alexithymia: a meta-analysis of emotion processing studies. NEUROSCIENCE AND BIOBEHAVIORAL REVIEWS, V37, P12 DOI 10.1016/j.neubiorev.2013.07.008	95	11
1	TAYLOR, GJ (1988) Criterion validity of the toronto alexithymia scale. PSYCHOSOMATIC MEDICINE, V50, P10 DOI 10.1097/00006842-198809000-00006	251	16
1	PARKER, JDA (1991) Alexithymia and depression - distinct or overlapping constructs. COMPREHENSIVE PSYCHIATRY DOI 10.1016/0010-440X(91)90015-5	167	6
1	KLEIGER, JH (1980) The development of an mmpi alexithymia scale. PSYCHOTHERAPY AND PSYCHOSOMATICS DOI 10.1159/000287442	122	16
1	KRYSTAL, JH (1986) Assessment of alexithymia in posttraumatic-stress-disorder and somatic illness - introduction of a reliable measure. PSYCHOSOMATIC MEDICINE, V48, P11 DOI 10.1097/00006842-198601000-00007	120	3

1	Luminet, O (1999) Relation between alexithymia and the five-factor model of personality: a facet-level analysis. JOURNAL OF PERSONALITY ASSESSMENT, V73, P14 DOI 10.1207/S15327752JPA7303_4	116	7
1	PARKER, JDA (1989) The alexithymia construct - relationship with sociodemographic variables and intelligence. COMPREHENSIVE PSYCHIATRY DOI 10.1016/0010-440X(89)90009-6	116	3
1	BAGBY, RM (1988) Alexithymia - a comparative-study of 3 self-report measures. JOURNAL OF PSYCHOSOMATIC RESEARCH, V32, P10 DOI 10.1016/0022-3999(88)90094-3	115	18
1	TAYLOR, GJ (1990) Validation of the alexithymia construct - a measurement-based approach. CANADIAN JOURNAL OF PSYCHIATRY-REVUE CANADIENNE DE PSYCHIATRIE DOI 10.1177/070674379003500402	113	3
1	HAVILAND, MG (1988) Validation of the toronto alexithymia scale with substance abusers. PSYCHOTHERAPY AND PSYCHOSOMATICS DOI 10.1159/000288104	98	13
1	Roedema, TM (1999) Emotion-processing deficit in alexithymia. PSYCHOPHYSIOLOGY DOI 10.1017/S0048577299980290	98	9
2	Taylor, GJ (2000) Recent developments in alexithymia theory and research. CANADIAN JOURNAL OF PSYCHIATRY-REVUE CANADIENNE DE PSYCHIATRIE DOI 10.1177/070674370004500203	394	8
2	Honkalampi, K (2000) Depression is strongly associated with alexithymia in the general population. JOURNAL OF PSYCHOSOMATIC RESEARCH DOI 10.1016/S0022-3999(99)00083-5	360	3
2	Parker, JDA (2003) The 20-item toronto alexithymia scale - iii. reliability and factorial validity in a community population. JOURNAL OF PSYCHOSOMATIC RESEARCH DOI 10.1016/S0022-3999(02)00578-0	343	3
2	Salminen, JK (1999) Prevalence of alexithymia and its association with sociodemographic variables in the general population of finland. JOURNAL OF PSYCHOSOMATIC RESEARCH DOI 10.1016/S0022-3999(98)00053-1	283	3
2	Vorst, HCM (2001) Validity and reliability of the bermond-vorst alexithymia questionnaire. PERSONALITY AND INDIVIDUAL DIFFERENCES, V30, P22 DOI 10.1016/S0191-8869(00)00033-7	283	2
2	TAYLOR, GJ (1988) Criterion validity of the toronto alexithymia scale. PSYCHOSOMATIC MEDICINE, V50, P10 DOI 10.1097/00006842-198809000-00006	251	4
2	Kooiman, CG (2002) The assessment of alexithymia - a critical review of the literature and a psychometric study of the toronto alexithymia scale-20. JOURNAL OF PSYCHOSOMATIC RESEARCH DOI 10.1016/S0022-3999(02)00348-3	231	6
2	Lumley, MA (1996) How are alexithymia and physical illness linked? a review and critique of pathways. JOURNAL OF PSYCHOSOMATIC RESEARCH, V41, P14 DOI 10.1016/S0022-3999(96)00222-X	213	2
2	HENDRYX, MS (1991) Dimensions of alexithymia and their relationships to anxiety and depression. JOURNAL OF PERSONALITY ASSESSMENT, V56, P11 DOI 10.1207/s15327752jpa5602_4	208	3
2	PARKER, JDA (1993) Factorial validity of the 20-item toronto alexithymia scale. EUROPEAN JOURNAL OF PERSONALITY DOI 10.1002/per.2410070403	200	2

3	Taylor, GJ (2000) Recent developments in alexithymia theory and research. CANADIAN JOURNAL OF PSYCHIATRY-REVUE CANADIENNE DE PSYCHIATRIE DOI 10.1177/070674370004500203	394	2
3	Lane, RD (2000) Pervasive emotion recognition deficit common to alexithymia and the repressive coping style. PSYCHOSOMATIC MEDICINE, V62, P10 DOI 10.1097/00006842-200007000-00007	201	2
3	Harrison, Amy (2009) Emotion recognition and regulation in anorexia nervosa. CLINICAL PSYCHOLOGY & PSYCHOTHERAPY DOI 10.1002/cpp.628	185	2
3	Herbert, Beate M (2011) On the relationship between interoceptive awareness and alexithymia: is interoceptive awareness related to emotional awareness?. JOURNAL OF PERSONALITY, V79, P27 DOI 10.1111/j.1467-6494.2011.00717.x	181	9
3	Bird, G (2013) Mixed emotions: the contribution of alexithymia to the emotional symptoms of autism. TRANSLATIONAL PSYCHIATRY DOI 10.1038/tp.2013.61	180	6
3	Svaldi, Jennifer (2012) Emotion regulation deficits in eating disorders: a marker of eating pathology or general psychopathology?. PSYCHIATRY RESEARCH DOI 10.1016/j.psychres.2011.11.009	180	2
3	Grynberg, Delphine (2012) Alexithymia and the processing of emotional facial expressions (efes): systematic review, unanswered questions and further perspectives. PLOS ONE DOI 10.1371/journal.pone.0042429	122	6
3	Zeidner, Moshe (2012) The emotional intelligence, health, and well-being nexus: what have we learned and what have we missed?. APPLIED PSYCHOLOGY-HEALTH AND WELL BEING DOI 10.1111/j.1758-0854.2011.01062.x	121	3
3	Bird, Geoffrey (2014) The self to other model of empathy: providing a new framework for understanding empathy impairments in psychopathy, autism, and alexithymia. NEUROSCIENCE AND BIOBEHAVIORAL REVIEWS, V47, P13 DOI 10.1016/j.neubiorev.2014.09.021	120	2
3	Grynberg, Delphine (2010) Alexithymia in the interpersonal domain: a general deficit of empathy?. PERSONALITY AND INDIVIDUAL DIFFERENCES DOI 10.1016/j.paid.2010.07.013	103	2
4	Bernhardt, Boris C (2012) The neural basis of empathy. ANNUAL REVIEW OF NEUROSCIENCE, VOL 35 Annual Review of Neuroscience, V35, P23 DOI 10.1146/annurev-neuro-062111-150536	405	3
4	Bird, G (2013) Mixed emotions: the contribution of alexithymia to the emotional symptoms of autism. TRANSLATIONAL PSYCHIATRY DOI 10.1038/tp.2013.61	180	3
4	Vellante, Marcello (2013) The "reading the mind in the eyes" test: systematic review of psychometric properties and a validation study in italy. COGNITIVE NEUROPSYCHIATRY, V18, P29 DOI 10.1080/13546805.2012.721728	154	3
4	Grynberg, Delphine (2012) Alexithymia and the processing of emotional facial expressions (efes): systematic review, unanswered questions and further perspectives. PLOS ONE DOI 10.1371/journal.pone.0042429	122	2
4	McDonald, Skye (2013) Impairments in social cognition following severe traumatic brain injury. JOURNAL OF THE INTERNATIONAL NEUROPSYCHOLOGICAL SOCIETY, V19, P16 DOI 10.1017/S1355617712001506	91	2

4	Garfinkel, Sarah N (2016) Discrepancies between dimensions of interoception in autism: implications for emotion and anxiety. BIOLOGICAL PSYCHOLOGY, V114, P10 DOI 10.1016/j.biopsycho.2015.12.003	86	3
4	Lockwood, Patricia L (2013) Dissecting empathy: high levels of psychopathic and autistic traits are characterized by difficulties in different social information processing domains. FRONTIERS IN HUMAN NEUROSCIENCE DOI 10.3389/fnhum.2013.00760	84	2
4	Ferguson, Eamonn (2013) Personality is of central concern to understand health: towards a theoretical model for health psychology. HEALTH PSYCHOLOGY REVIEW DOI 10.1080/17437199.2010.547985	80	2
4	Shah, Punit (2016) Alexithymia, not autism, is associated with impaired interoception. CORTEX DOI 10.1016/j.cortex.2016.03.021	77	6
4	Strigo, Irina A (2013) Altered insula activation during pain anticipation in individuals recovered from anorexia nervosa: evidence of interoceptive dysregulation. INTERNATIONAL JOURNAL OF EATING DISORDERS, V46, P11 DOI 10.1002/eat.22045	75	2
5	TAYLOR, GJ (1992) The revised toronto alexithymia scale - some reliability, validity, and normative data. PSYCHOTHERAPY AND PSYCHOSOMATICS DOI 10.1159/000288571	186	3
5	Honkalampi, K (2000) Is alexithymia a permanent feature in depressed patients? results from a 6-month follow-up study. PSYCHOTHERAPY AND PSYCHOSOMATICS DOI 10.1159/000012412	108	2
5	Honkalampi, K (1999) Factors associated with alexithymia in patients suffering from depression. PSYCHOTHERAPY AND PSYCHOSOMATICS DOI 10.1159/000012343	105	3
5	Fava, GA (2000) Psychosomatic medicine: emerging trends and perspectives. PSYCHOTHERAPY AND PSYCHOSOMATICS, V69, P14 DOI 10.1159/000012393	100	4
5	Jula, A (1999) Alexithymia - a facet of essential hypertension. HYPERTENSION DOI 10.1161/01.HYP.33.4.1057	98	2
5	Fava, GA (2001) Assessment of psychological distress in the setting of medical disease. PSYCHOTHERAPY AND PSYCHOSOMATICS DOI 10.1159/000056249	72	3
5	Porcelli, P (2000) Assessing somatization in functional gastrointestinal disorders: integration of different criteria. PSYCHOTHERAPY AND PSYCHOSOMATICS DOI 10.1159/000012394	60	3
5	HENRY, JP (1992) Shared neuroendocrine patterns of posttraumatic-stress-disorder and alexithymia. PSYCHOSOMATIC MEDICINE DOI 10.1097/00006842-199207000-00003	56	3
5	Jones, MP (2004) Alexithymia and somatosensory amplification in functional dyspepsia. PSYCHOSOMATICS DOI 10.1176/appi.psy.45.6.508	38	2
5	Porcelli, P (2001) Criterion-related validity of the diagnostic criteria for psychosomatic research for alexithymia in patients with functional gastrointestinal disorders. PSYCHOTHERAPY AND PSYCHOSOMATICS DOI 10.1159/000056251	35	3

Supplementary Table 6 List of the most influential cited documents within the 5 biggest clusters obtained via Document Co-citation Analyses (DCA).

Cluster # and label by LLR	Citations Freq	Burst	Centrality	Sigma	Cited Document	Year
**#0 Personality Disorder**	232	39.53	0.05	2.9	**AmericanPsychiatricAssociation	1994
	317	32.31	0.04	3.34	Taylor GJ	2004
	785	29.98	0.03	2.63	Taylor G J	1997
	67	26.06	0	1.16	Li SW	2015
	66	23.33	0.01	1.2	Ogrodniczuk JS	2011
	88	23.09	0.01	2.14	Lane RD	1998
	51	21.36	0	1.1	Joukamaa M	2001
	71	21.32	0.01	1.54	Grabe HJ	2004
	43	20.88	0.01	1.76	Luminet O	1999
	157	20.57	0.04	3.18	Lumley MA	1996
	69	20.28	0.01	1.77	Sifneos PE	1996
	1941	-	0.04	1	BAGBY RM	1994
	1161	4.06	0.02	1.1	BAGBY RM	1994
**#1 mmpi alexithymia scale**	268	64.39	0.11	692.91	TAYLOR GJ	1984
	131	57.74	0.07	38.48	APFEL RJ	1979
	111	37.35	0.06	7.81	KRYSTAL H	1979
	73	36.89	0.04	3.98	KLEIGER JH	1980
	151	36.2	0.13	76.13	FREYBERGER H	1977
	78	35.79	0.05	4.89	LESSER IM	1981
	48	27.82	0.02	1.68	BLANCHARD	1981
	105	33.68	0.06	7.53	SIFNEOS PE	1972
	1010	-	0.14	-	SIFNEOS PE	1973
**#2 toronto alexithymia scale**	413	70.11	0.07	151.25	TAYLOR GJ	1985
	143	54.25	0.03	5.94	TAYLOR GJ	1988
	97	49.15	0.03	3.41	BAGBY RM	1986
	73	38.07	0.01	1.43	BAGBY RM	1988
	58	33.59	0.01	1.24	TAYLOR GJ	1990
	100	31.54	0.04	3.77	NEMIAH JC	1970
	130	30.4	0.03	2.23	PARKER JDA	1993
	59	29.47	0.03	2.15	TAYLOR GJ	1992
	112	28.27	0.03	2.48	KRYSTAL H	1988
	323	27.54	0.09	10.52	TAYLOR GJ	1991

**#3 toronto alexithymia scale**						
**(renamed "Emotional Information processing")**	137	37.43	0.03	2.58	Gratz KL	2004
	98	27.78	0.01	1.29	Grynberg D	2012
	72	21.69	0.01	1.32	van der Velde J	2013
	53	21.56	0	1.08	Hayes A F	2013
	64	21.46	0.01	1.12	Taylor G J	1999
	91	18.94	0.02	1.34	Swart M	2009
	123	18.57	0.03	1.63	BARON RM	1986
	73	18.4	0.01	1.2	DAVIS MH	1983
	118	18.26	0.01	1.31	Gross JJ	2003
	58	18.26	0.01	1.14	Reker M	2010
	65	18.12	0.02	1.42	Grynberg D	2010
	121	17.44	0.02	1.42	Kano M	2003
	143	17.42	0.02	1.45	Berthoz S	2002
	37	17.17	0.01	1.14	Moriguchi Y	2013
	64	16.81	0.02	1.47	Bydlowski S	2005
	91	16.27	0.04	1.81	Moriguchi Y	2007
	203	4.11	0.08	1.39	LANE RD	1987
	107	13.85	0.05	1.86	PARKER JDA	1993
	235	10.18	0.04	1.55	Lane RD	1996
**#4 autism spectrum disorder**	204	58.38	0.01	1.71	**AmericanPsychiatricAssociation	2013
	95	31.9	0.02	1.93	Bird G	2013
	95	31.9	0.01	1.22	Herbert BM	2011
	143	28.67	0.01	1.53	Bird G	2010
	89	27.32	0.01	1.17	Cook R	2013
	42	21.55	0.01	1.2	Brewer R	2016
	83	20.93	0.01	1.15	Baron-Cohen S	2001
	46	19.58	0	1.06	Shah P	2016
	67	16.88	0.01	1.26	Craig AD	2009
	43	16.71	0.02	1.37	SCHANDRY R	1981
	80	16.64	0	1.08	Silani G	2008
	49	16.42	0.01	1.12	Craig AD	2002
	30	15.39	0	1	Garfinkel SN	2015
	29	14.87	0	1.07	Westwood H	2017

	57	14.35	0	1.03	Berthoz S	2005
	68	6.69	0.08	1.72	HAMILTON M	1960
**#5 psychological factor**	27	13.76	0.01	1.08	Honkalampi K	1999
	22	12.41	0.01	1.07	Porcelli P	1999
	15	9.18	0	1.03	KAUHANEN J	1992
	13	7.22	0.01	1.05	Verissimo R	1998
	18	7.2	0	1.01	Todarello O	1995
	12	7.11	0	1.04	De Berardis D	2008
	12	7	0.01	1.05	Kooiman CG	1998
	22	6.65	0	1.01	Beck AT	1996
	11	6.41	0	1.01	Porcelli P	2001
	10	6.1	0.01	1.08	LIPOWSKI ZJ	1988
	9	5.97	0	1.01	PARKER JDA	1991
	32	5.35	0.03	1.16	FAVA GA	1995
	8	5.04	0	1	BAGBY RM	1991
	8	4.87	0	1	Infrasca R	1997
